# Comprehensive Analysis of N6-methyladenosine Modification Patterns Associated With Multiomic Characteristics of Bladder Cancer

**DOI:** 10.3389/fmed.2021.757432

**Published:** 2021-12-23

**Authors:** Jingchao Liu, Jianlong Wang, Meng Wu, Wei Zhang, Lingfeng Meng, Jiawen Wang, Zhengtong Lv, Haoran Xia, Yaoguang Zhang, Jianye Wang

**Affiliations:** ^1^Department of Urology, Beijing Hospital, National Center of Gerontology, Institute of Geriatric Medicine, Chinese Academy of Medical Sciences, Beijing, China; ^2^Graduate School of Peking Union Medical College, Chinese Academy of Medical Sciences, Beijing, China

**Keywords:** N6-methyladenosine, m6A, tumor mutation burden, bladder tumor, immunotherapy

## Abstract

**Purpose:** To comprehensively analyze N6-methyladenosine modification patterns in bladder tumors and to further systematically explore the inherent relationships between these modification patterns and multiomic tumor characteristics.

**Materials and Methods:** A total of 901 bladder tumor samples, including 405 samples from TCGA database, 188 samples from GSE13507 and 308 samples from GSE32894, were included in this systematic analysis. The N6-methyladenosine modification patterns were identified utilizing unsupervised clustering analysis. To quantify N6-methyladenosine modification patterns, the m6Ascore of individual sample was developed using principal component analysis algorithms. Relationships among immune infiltration, tumor mutation burden, various clinical characteristics, molecular subtypes, and the m6Ascore were systematically analyzed. The guiding value of m6Ascore in immunotherapy was further validated in an external trial cohort. Genomics of Drug Sensitivity in Cancer expression references were also utilized to perform drug sensitivity analysis for patients with distinct m6A modification patterns.

**Results:** We determined three different N6-methyladenosine modification patterns for 901 bladder tumors. The quantitative m6Ascore of individual sample derived from N6-methyladenosine modification patterns could play a significant role in predicting overall survival, immune cell infiltration, and classic oncogene mutations. A low m6Ascore combined with high tumor mutation burden indicated better survival outcomes (*p* < 0.001). A higher m6Ascore also indicated a higher grade, higher T and N stage, elder ages, higher death rate, and higher PD1/PDL1/CTLA4 expressions (*p* < 0.01). The Basal type tended to exhibit significantly higher m6Ascores than the Luminal and Neuronal subtypes. External immunotherapy cohorts demonstrated that no difference in therapeutic effects was noted between the high and low m6Ascore groups when anti-PD1 immunotherapy was exclusively administered. When anti-PD1 and anti-CTLA4 immunotherapy were simultaneously administered, the high m6Ascore group had a significantly better prognosis than the low m6Ascore group (*p* < 0.001). High m6A groups were potentially sensitive to various medical treatments including Bleomycin, Bortezomib, Cisplatin, Cyclopamine, Dasatinib, Docetaxe, Rapamycin, and Vinblastine in this study.

**Conclusions:** This study systematically revealed the important roles of m6A methylation modification patterns in bladder tumors. Detailed quantification of m6A modification patterns could improve our understanding of the bladder tumor microenvironments and could provide guidance for future immunotherapy strategies.

## Introduction

Bladder cancer was viewed as the most common malignant cancer originating from the urinary tract and had caused >200 thousand deaths worldwide (1). The global incidence of bladder cancer in males was ~4-fold higher than that in females (2). Various characteristics, including muscle invasive/non-invasive status and pathological grade, were currently utilized to guide treatment strategies and patient prognosis. The pathogenesis mechanism for bladder cancer was rather complex, and more detailed biological studies were urgently needed to improve the current prognosis. The classical oncogene mutations responsible for bladder cancer included TP53, RB1, ERCC2, and FGFR3 (3). Cisplatin-based chemotherapy was suggested as the first-line treatment for advanced bladder tumors, and immunotherapy was also suggested as a second-line treatment (4). However, long-term responses to current therapies were rare, and tumors often relapsed within the next 5 years due to the limited understanding of their progressive mechanism.

DNA and histone proteins have been reported to play a regulatory function in gene expression through reversible epigenetic modifications in recent decades (5). In recent years, N6-methyladenosine (m6A) modification was also identified as the first reversible RNA methylation pattern, and m6A activity was noted in polyadenylated mRNAs and various long non-coding RNAs (6). Various tumor types and corresponding immune responses have been reported to exhibit a close relationship with abnormal m6A activity (7, 8). The m6A methylation process depended on the expression levels of methyltransferases and demethylases, which were also called “writers” and “erasers,” respectively. In addition, binding proteins, which were also called “readers,” were also important for m6A biological functions (9). Methyltransferases included METTL14, WTAP, RBM15, ZC3H13, METTL3, and so on. Demethylases included ALKBH5 and FTO. The “readers” included YTHDF1/2/3, YTHDC1/2, HNRNPA2B1, LRPPRC, and so on (10). Three components interacted systematically to catalyze the biological activities of the m6A methylation process. Increasing studies had also confirmed that abnormal expression changes in m6A methylation regulators were significantly related to the tumor immune microenvironment (11). The tumor microenvironment played a crucial role in tumor progression and patient survival outcomes. Exploring the characteristics of the microenvironment and further predicting the response to immunotherapy have become promising research directions in recent years (12). Increasing evidence has shown a close relationship between m6A methylation and the immune microenvironment. Abnormal expression levels of m6A methylation regulators significantly affected immune cell infiltration. The overexpression or inhibition of m6A regulators had also been reported to predict immunotherapy results in various tumor types (13). Yu et al. identified that ALKBH5 overexpression limited bladder cancer progression and promoted bladder cancer cells sensitivity to cisplatin in a CK2-mediated m6A-dependent manner (14). Yang et al. also reported that METTL3 contributed to the development and progression of bladder cancer (15). Cheng et al. also confirmed that the tumor-promoting role of METTL3 occurred through the AFF4/NF-kB/Myc signaling pathway in bladder cancer (16).

Nevertheless, all of the above studies exclusively focused on the roles of only one or two m6A regulators in a limited number of bladder tumors due to sequencing technology limitations at that time. The antitumor mechanism of m6A methylation must include various regulators that are closely connected and interact with each other throughout the entire process of bladder cancer progression. Comprehensive analysis of m6A methylation patterns and their relationship with the tumor microenvironment could provide considerable information for advanced therapy strategies targeting bladder cancer. In this study, we comprehensively analyzed the m6A methylation modification patterns of 901 bladder tumor samples on the basis of reported m6A methylation regulators and further systematically explored the inherent relationship between these modification patterns and multiomic characteristics of bladder tumors. We determined three different m6A modification patterns and successfully quantified m6A modifications using the m6Ascore. We surprisingly revealed that m6A modification levels significantly correlated with patient overall survival, immune cell infiltration, classic oncogene mutation status, pathological grading, and patient response to immunotherapy. Only a combined immunotherapy strategy (anti-PD1 plus anti-CTLA4) had a significant effect in the high m6Ascore group.

## Materials and Methods

### Data Collection and Processing

We downloaded the open expression matrix of mRNA and corresponding clinical files of bladder tumor samples from The Cancer Genome Atlas (TCGA) public database and the Gene Expression Omnibus (GEO) database. All the samples with unavailable clinical files were excluded from further analysis. A total of 901 bladder tumor samples, including 405 tumor samples from TCGA database, 188 tumor samples from the GEO database (GSE13507) and an additional 308 bladder tumors from the GEO database (GSE32894), were involved in this systematic analysis. Only TCGA datasets with available gene copy number variation and gene mutation information were used for mutation distribution analysis of the m6A modification regulators. If the public microarray resources were from the Affymetrix platform, then initial “CEL” documents were first downloaded, and background adjustment and matrix normalization were further performed with “affy” packages. If the open resources were from other platforms, then the normalized sources were directly downloaded. The FPKM files were firstly downloaded from TCGA public database and then translated into transcripts per kilobase million files using R software. The aim of the above data processing was to maintain consistency and comparability between TCGA and different GEO datasets.

### Predictive Analysis and Unsupervised Clustering for 10 m6A Regulators in Merged Datasets

Due to different expression platforms between different series, a total of 10 m6A modulation regulators were finally identified from the merged datasets, including TCGA, GSE13507, and GSE32894 datasets. The 10 identified m6A regulators included ALKBH5, RBM15, RBM15B, YTHDC1, YTHDC2, YTHDF1, YTHDF3, IGFBP2, IGFBP3, and RBMX. Univariate Cox regression analysis was performed to explore the predictive role of m6A regulators in survival. To explore the detailed m6A modification patterns in the large cohort, we adopted the unsupervised clustering method to classify the detailed modification patterns of bladder tumors. A consensus clustering strategy was utilized to determine the optimal cluster number and cluster stability (17). All clustering processes were performed by the ConsensuClusterPlus package in R software.

### GSVA Analysis, Differentially Expressed Genes (DEGs), Gene Ontology Analysis, and ssGSEA Among Three Different m6A Modification Patterns

To explore the internal difference of biological functions between three different m6A modification patterns, GSVA (Gene set variation analysis) was performed by the “GSVA” packages utilizing R software. The gene reference files named “c2.cp.kegg.v7.4.symbols” were available from the MSigDB database. The DEGs among the three m6A modification patterns were analyzed for the merged cohort (|logFC| > 1 and adjusted *p* < 0.01). DEGs were determined by applying the empirical Bayesian approach using the “limma” package. Gene Ontology analysis was utilized through R software with the “clusterProfiler” package. The gene sets associated with various immune cells, including activated CD8 T cells, regulatory T cells, and macrophages, were derived from previous studies (18). The ssGSEA was then performed to quantify the concentration of infiltration of various immune cells in the merged cohort utilizing the “gsva” package of R software (19).

### Development of m6A Gene Clusters and m6Ascore to Quantify Individual m6A Modification Patterns

We first identified the intersecting DEGs among three different m6A modification patterns. The intersecting DEGs were then included in prognostic value analysis for patient overall survival using univariate Cox regression methods. The prognostic intersecting DEGs were then included in the next analysis procedure. An unsupervised clustering method depending on the expression of the above genes was performed to classify patients into several different clusters, which we called m6A gene clusters. The amount and stability of m6A gene clusters were determined using the consensus clustering algorithm, and three distinct m6A gene clusters were finally identified in the study. The significant differences or prognostic value of the above prognostic intersecting DEGs could be illustrated by differential analysis among the three m6A gene clusters. The differences in overall survival and the expression levels of different m6A regulators among the three m6A gene clusters were also explored using Kaplan-Meier analysis and Student's *t*-test, respectively.

Then, we developed a quantification method to estimate the m6A modification patterns of individual samples from the study. We named this quantifying term m6Ascore for bladder tumor. Based on the expression of the above prognostic intersecting DEGs in the study, principal component analysis (PCA) was utilized to develop the detailed m6Ascore value. The m6Ascore was derived from both principal component 1 and principal component 2. This method can increase the weight coefficient of genes significantly related to other genes and reduce the influence of genes not related to other genes (20). The detailed quantification algorithm for the m6Ascore was as follows: m6Ascore value = ∑ (principal component 1_i_+ principal component 2_i_), where i indicates the expression of prognostic intersecting DEGs in the study. Then, the high and low m6Ascore groups were determined depending on the median m6Ascore value in the study.

### Exploration of the Correlation Between the m6Ascore and Survival Status, m6A Clusters, m6A Gene Clusters, and Immune Cell Infiltration

We compared overall survival status between the low and high m6Ascore groups utilizing Kaplan-Meier analysis combined with the log-rank test. The relationship among m6Ascore and m6A clusters, m6A gene clusters and dead/alive status was illustrated using a Sankey diagram for the study. The correlation between the m6A score and immune cell infiltration level was analyzed to further reveal the intrinsic biological function of the m6A regulators. The differences in the m6Ascore between three distinct m6A clusters and three distinct m6A gene clusters were also compared. Furthermore, ESTIMATE algorithm was utilized to evaluate different immune microenvironments for bladder cancers with distinct m6Ascore groups (21). The “corrplot,” “limma,” and “ggpubr” packages in R software were utilized for the above analysis.

### The Correlation Between the m6Ascore and Mutation Status of 8 Classic Oncogenes in the Large Cohort Study

To further investigate the molecular mechanism of m6A modification in bladder cancer, the correlation between the m6Ascore and previously reported oncogenes was evaluated in this study. The 8 selected classical genes with available mutation information from TCGA database included TP53, RB1, ERCC2, ATM, EP300, FGFR3, ELF3, and ERBB2 (22). Correlations of the tumor mutation burden (TMB) and m6Ascores were also explored by “ggpubr” and “reshape2” packages in R. Furthermore, the prognostic roles for survival outcomes of high/low TMB and high/low m6Ascore were also tested using Kaplan-Meier analysis in this large cohort study.

### Correlation of m6Ascore With Various Clinical Characteristics of Bladder Cancer

The chi-squared test was performed to compare the different ratio of tumor grade, clinical “T” and “N” stages, patient ages, and gender in the high/low m6Ascore groups. The difference of m6Ascores in the above subgroups was also compared using Student's *t*-test. Increasing studies have reported the feasibility and necessity of molecular subtype differentiation of bladder tumors in recent years (22). To further explore the basic mechanism for m6A modification and to confirm the feasibility for reported molecular subtype differentiation in recent years, the “ggpubr,” “ggplot2,” and “limma” packages of R software were also used to estimate the correlation between the m6Ascore and reported molecular subtypes of bladder cancer tumors in the study. Furthermore, the predictive ability for survival outcomes of m6Ascore in this study was also tested by the hierarchical classification of “G1/G2/G3,” “Ta-T1/T2-T4,” “Age>65/Age ≤ 65,” and “Female/Male” using Kaplan-Meier analysis.

A good quantification tool for bladder tumor patients should illustrate a consistent predictive role in different hierarchical classifications. The predictive abilities of various clinical parameters including m6Ascore risks, tumor grade, clinical stage and patients ages were also further compared by receiver operating characteristic (ROC) analysis.

### Potential Application of the m6Ascore to Guide Immunotherapy Strategy Choice

We extracted genomic PD-L1, PD-1, and CTLA-4 expression data from the merged gene matrix in our study. Standard gene names of PD-L1, PD-1, and CTLA-4 (CD274, PDCD1, and CTLA-4, respectively) were searched using the NCBI website (https://www.ncbi.nlm.nih.gov/gene). The “limma” and “ggpubr” packages in R were used to analyze expression differences of immune checkpoint genes in the high and low m6Ascore groups. Furthermore, the immunotherapeutic cohort from http://tcia.at/ was further used to validate the relationship between the m6Ascore and immunotherapeutic response. Four immunotherapy strategies, including the anti-CTLA-4 treatment strategy alone, the anti-PD-1 treatment strategy alone, the anti-CTLA-4 plus anti-PD-1 treatment strategy and no immunotherapy strategy, were evaluated in the high and low m6Ascore groups in this study. Additionally, Genomics of Drug Sensitivity in Cancer (GDSC) expression references were also utilized to investigate potential sensitive drugs for patients with high m6Ascore group (23). The half maximal inhibitory concentration (IC50) values were used in such drug sensitivity analysis (24).

### Statistical Analysis

The DEGs between tumor and non-tumor samples were determined by Student's *t*-test. The chi-squared test was used to compare categorical variables in this study. The ssGSEA scores between the high/low score groups were compared using the Mann-Whitney test with the BH method. One-way ANOVA was utilized to analyze differences among three or more groups. Survival outcomes between different groups were compared by Kaplan-Meier analysis with the log-rank test. All of the data analyses in the current study were performed using R software or SPSS (V 23.0). Unless otherwise noted, a *p* < 0.05 from a two-tailed test indicated significance.

## Results

### Copy Number Variations (CNVs) and Gene Mutations in m6A Modification Regulators in Bladder Tumors

**Figure 1:**
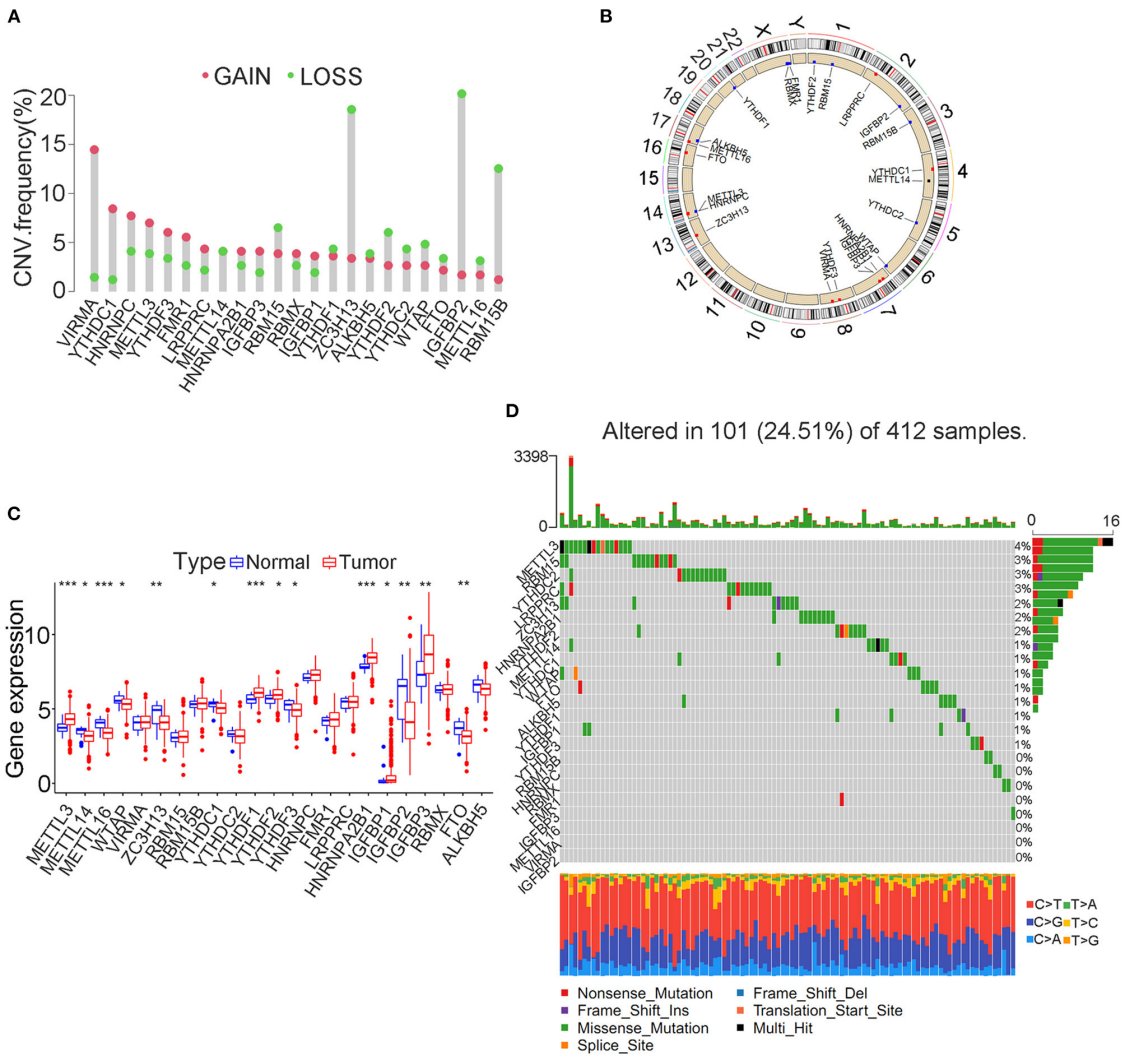
Landscape of genetic variation in m6A modification regulators in bladder tumors. **(A)** CNV distributions of m6A regulators in bladder tumors. **(B)** The CNV locations of m6A regulators on 23 human chromosomes. **(C)** Differences in the expression of m6A regulator genes between tumor and nontumor samples. **(D)** Waterfall plot illustrating genetic mutation of m6A regulators in bladder tumors. Individual patients are represented in each column. TMB is shown by upper bar plot. The frequency of genetic mutations in each m6A regulator is indicated by the number on the right. Each alteration type is shown in the right bar plot. The fraction of conversions is shown by the stacked bar plot below (*p < 0.05; **p < 0.01; ***p < 0.001).

The FPKM files were first downloaded from TCGA public database and then translated into transcripts per kilobase million files using R software. A total of 23 m6A modification regulators were identified from TCGA cohort with the available CNV and gene mutation files. The list of m6A modification regulators was illustrated in Supplementary Table 1. The CNV information of m6A regulators was shown in Supplementary Table 2. Figure 1A showed that two writers (VIRMA and METTL3) and most readers, including YTHDC1, YTHDF3, HNRNPC, FMR1, LRPPRC, HNRNPA2B1, IGFBP3, and IGFBP1, tended to obtain an amplification of CNV, whereas two erasers (FTO and ALKBH5) and most writers, including METTL16, WTAP, ZC3H13, RBM15, and RBM15B, tended to get a depletion of CNV in bladder tumors. Figure 1B further illustrated the detailed location of the above CNVs on human chromosomes. We also explored the expression differences in m6A regulators between bladder tumor and non-tumor samples. Figure 1C showed that there was a significant difference in m6A modification regulators between bladder tumor and non-tumor samples. The m6A regulators with CNV amplification, such as METTL3, YTHDF1, HNRNPC, HNRNPA2B1, and TGFBP3, tended to obtain higher expression levels in bladder tumor samples. The m6A regulators with CNV depletion during bladder tumor, such as METTL14, ZC3H13, METTL16, IGFBP2, FTO, and ALKBH5, tended to exhibit significantly low expression levels. Gene mutation information of TCGA cohort was also shown in Supplementary Table 3, and corresponding tumor mutation burden (TMB) information was also presented in Supplementary Table 4. The waterfall plot in Figure 1D detailed the whole gene mutations of m6A regulators in the bladder tumor samples. In total, 101 of 412 bladder tumor samples exhibited gene mutations in m6A regulators with a high mutation frequency of 24.51%. MTTL3, RBM15, YTHDC2, and LRPPRC were the m6A regulators with the highest mutation rates. METTL16, VIRMA, and IGFBP2 represented m6A regulators without gene mutations. These results indicated high heterogeneity in the gene expression levels and mutation status of m6A regulators in bladder cancer tumor samples. The complicated interactions among m6A modification regulators promoted the pathogenesis of bladder cancer through an interactive mechanism.

### Predictive Analysis of m6A Regulators and m6A Clusters in a Merged Cohort

**Figure 2:**
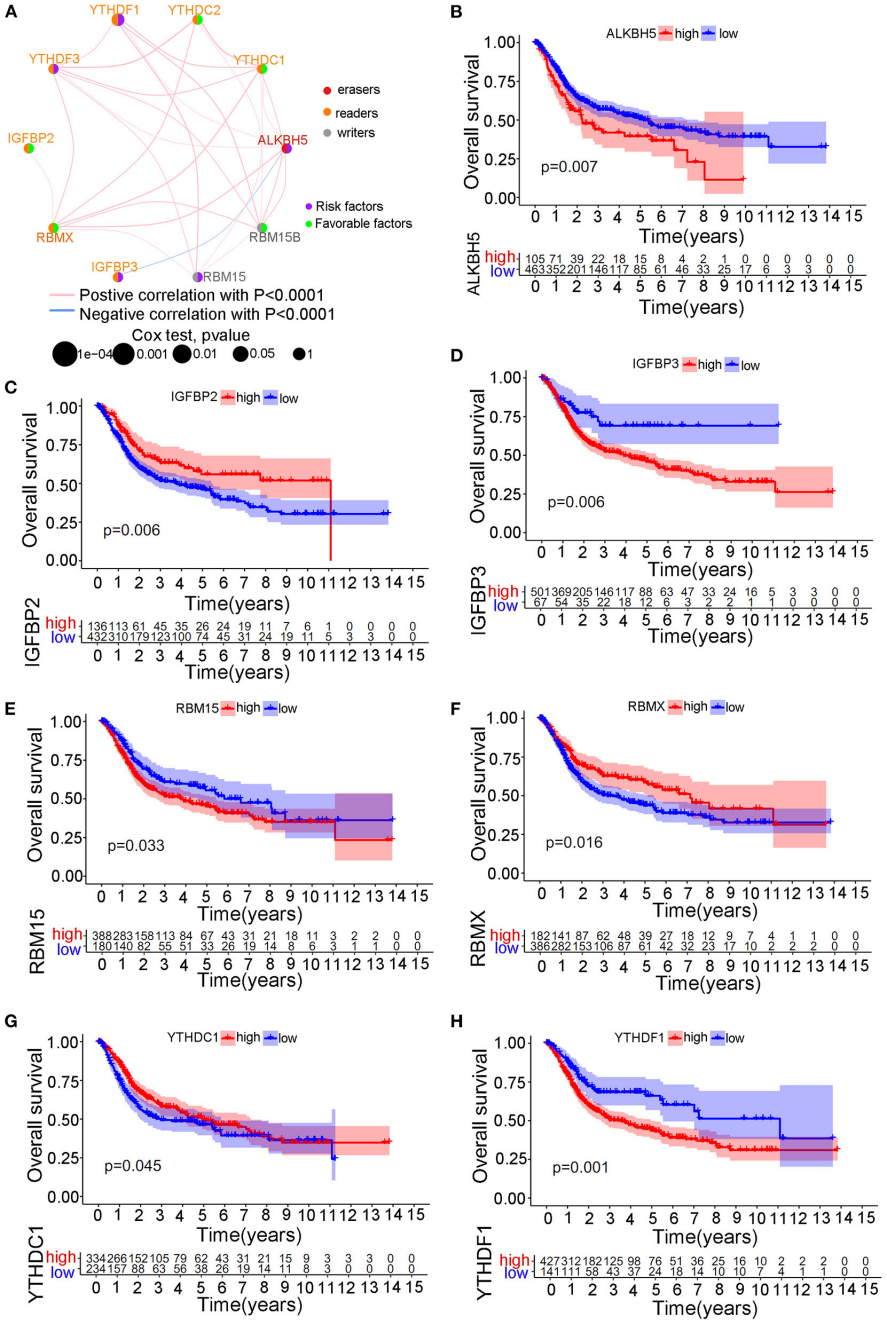
The survival prognostic roles of m6A regulator during the merged cohort. **(A)** The correlation network between different m6A regulators in bladder tumors. **(B)** Kaplan-Meier curve showing the prognostic value of ALKBH5 for survival in the merged cohort. **(C)** Kaplan-Meier curve showing the prognostic value of IGFBP2 for survival in the merged cohort. **(D)** Kaplan-Meier curve showing the prognostic value of IGFBP3 for survival in the merged cohort. **(E)** Kaplan-Meier curve showing the prognostic value of RBM15 for survival in the merged cohort. **(F)** Kaplan-Meier curve showing the prognostic value of RBMX for survival in the merged cohort. **(G)** Kaplan-Meier curve showing the prognostic value of YTHDC1 for survival in the merged cohort. **(H)** Kaplan-Meier curve showing the prognostic value of YTHDF1 for survival in the merged cohort.

We selected three cohorts including TCGA cohort, GSE13507 cohort and GSE32894 cohort with available transcriptome and clinical information to form one merged cohort to perform further analysis in this study. Despite the use of different matrix platforms for the three distinct cohorts, a total of 10 m6A modification regulators were finally identified from the matrix document, including RBM15, RBM15B, YTHDC1, YTHDC2, YTHDF1, YTHDF3, IGFBP2, IGFBP3, RBMX, and ALKBH5. Detailed expression data of the 10 m6A regulators in the study were shown in Supplementary Table 5. Univariate Cox regression analysis was performed to explore the predictive value of the 10 m6A regulators in bladder tumor samples. The regulator network was also developed to illustrate the interactions and relative connections among the m6A regulators (Figure 2A). We surprisingly found that 7 of 10 (70%) m6A modification regulators posed a significant predictive value for bladder tumor survival prognosis, indicating that the m6A modification played a vital role in bladder tumor development and progression. The prognostic m6A regulators included ALKBH5 (*p* < 0.01), IGFBP2 (*p* < 0.01), IGFBP3 (*p* < 0.01), RBM15 (*p* < 0.05), RBMX (*p* < 0.05), YTHDC1 (*p* < 0.05), and YTHDF1 (*p* < 0.01) (Table 1). Figures 2B–H shows the Kaplan-Meier curve analysis results concerning prognostic m6A regulators and overall survival in patients with bladder cancer.

**Table 1:** Prognostic analysis of m6A-related genes for survival status.

**Gene ID**	**HR**	**HR.95L**	**HR.95H**	***P*-value**
RBM15	1.22	0.95	1.56	**0.03**
RBM15B	0.99	0.77	1.26	0.18
YTHDC1	0.98	0.74	1.29	**0.04**
YTHDC2	0.91	0.72	1.14	0.08
YTHDF1	1.48	1.10	2.00	**<** **0.01**
YTHDF3	1.06	0.84	1.34	0.19
IGFBP2	0.96	0.88	1.03	**<** **0.01**
IGFBP3	1.08	1.00	1.17	**<** **0.01**
RBMX	0.84	0.65	1.08	**0.01**
ALKBH5	1.28	0.99	1.64	**<** **0.01**

*Bold values indicate statistically significant differences (p < 0.05)*.

**Figure 3:**
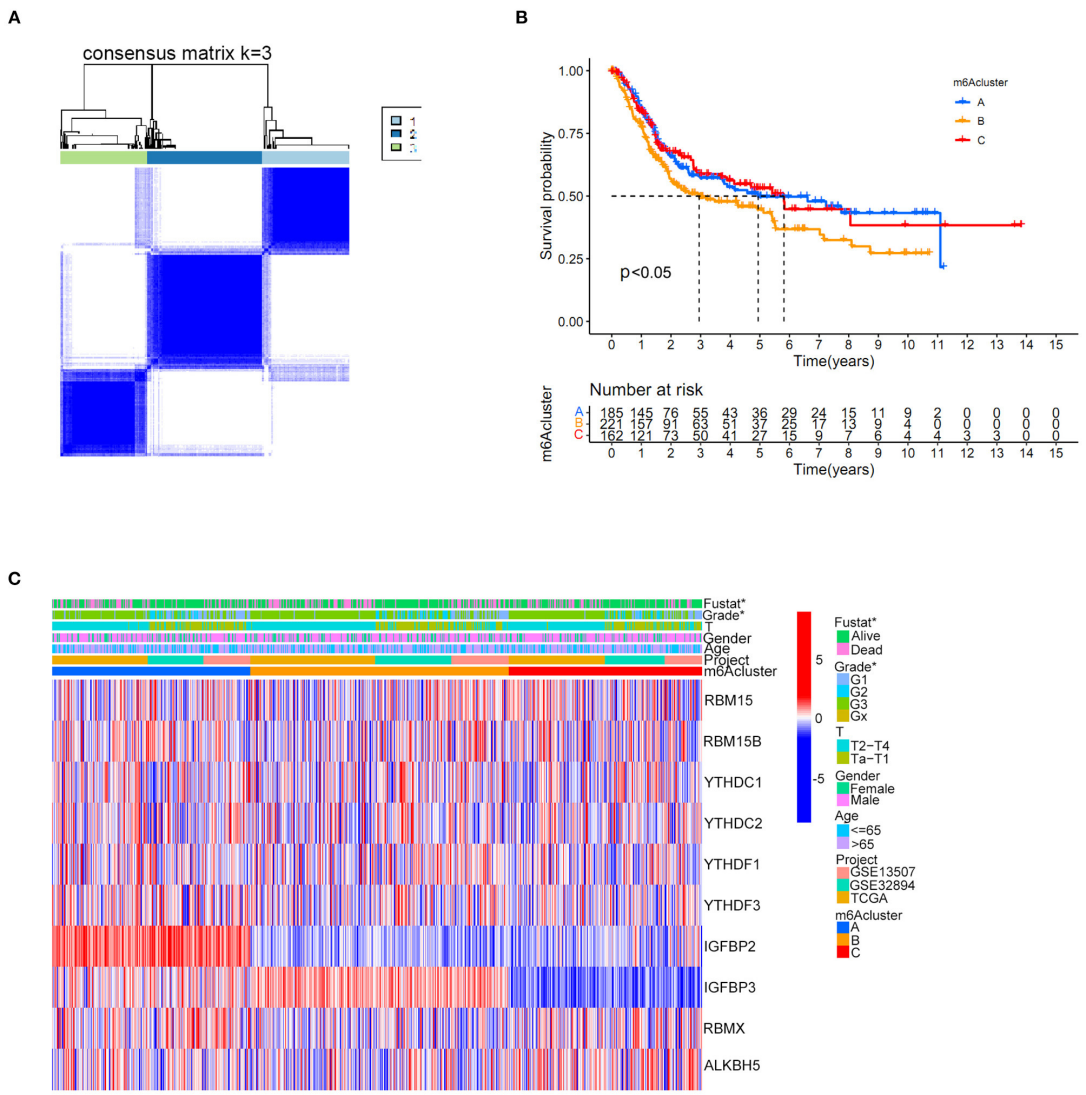
m6A cluster results in the merged cohort. **(A)** Consensus clustering matrix for *k* = 3. **(B)** Kaplan-Meier curve showing the survival prognostic value of three distinct m6A clusters. **(C)** Heatmap illustrating various clinicopathological features of three distinct m6A clusters (*p < 0.05).

Distinct m6A clusters were classified using the “ConsensusClusterPlus” package in R software based on the expression of 10 m6A regulators in the merged cohort. A total of three different m6A clusters were finally identified by unsupervised clustering with 268 samples in m6A cluster A, 361 samples in m6A cluster B and 272 samples in m6A cluster C (Figure 3A). Prognostic analysis identified significant survival differences among the three m6A clusters (Figure 3B). Detailed classified m6A cluster information and complete clinical information for the merged cohort were reported in Supplementary Tables 6, 7, respectively. Relationships between m6A cluster results and other clinical characteristics, including survival status, age, gender, Grade and T stage, were also directly demonstrated by the heatmap in Figure 3C. The m6A clusters were identified to pose significant relationships with tumor grades and patients survival status (Figure 3C).

### GSVA Analysis, Differentially Expressed Genes (DEGs), Gene Ontology (GO) Analysis, and ssGSEA Among Three Different m6A Modification Patterns

**Figure 4:**
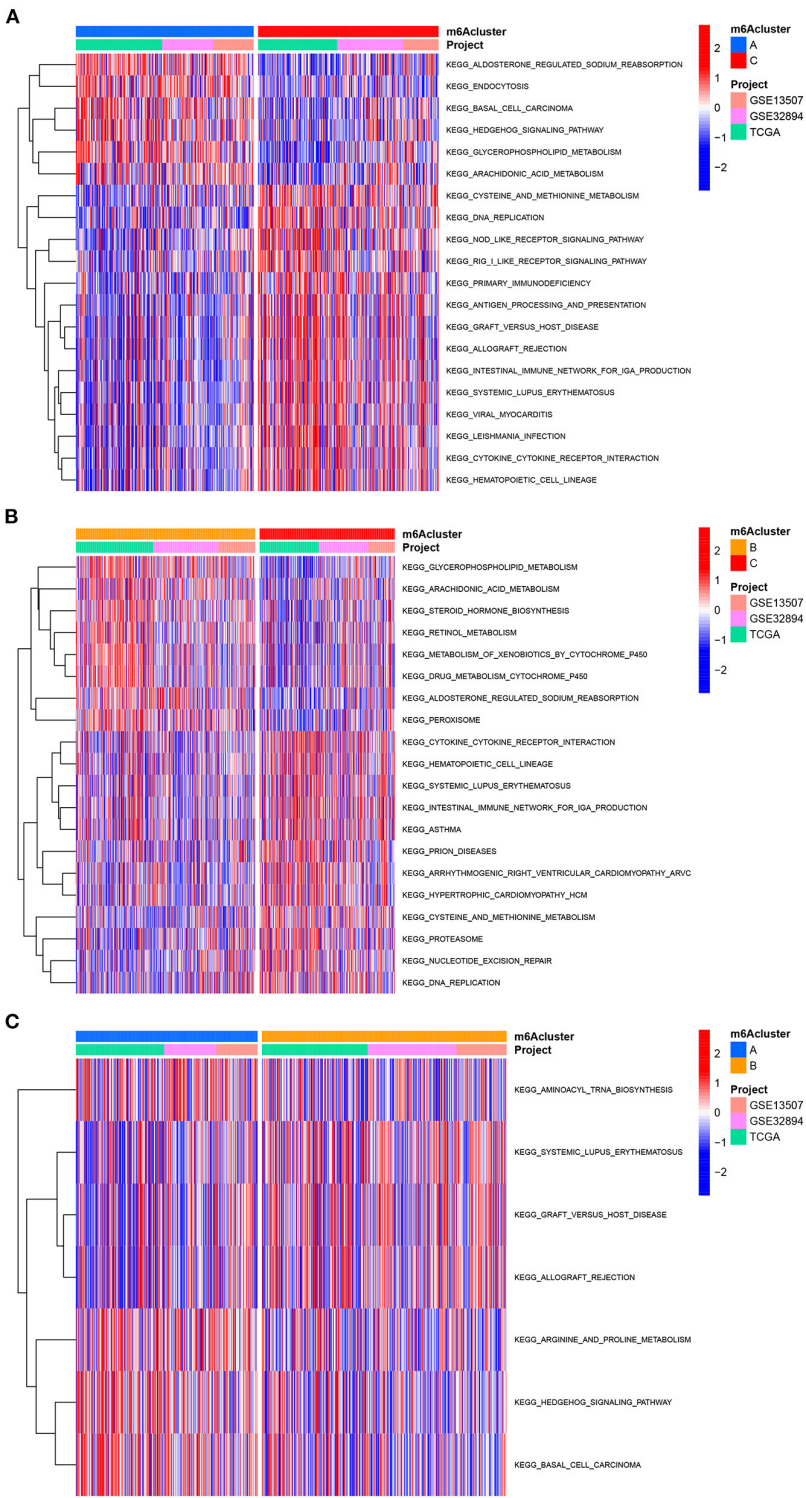
GSVA analysis among three m6A clusters in the merged cohort. **(A)** GSVA analysis between m6A cluster A and m6A cluster C. **(B)** GSVA analysis between m6A cluster B and m6A cluster C. **(C)** GSVA analysis between m6A cluster A and m6A cluster B.

**Figure 5:**
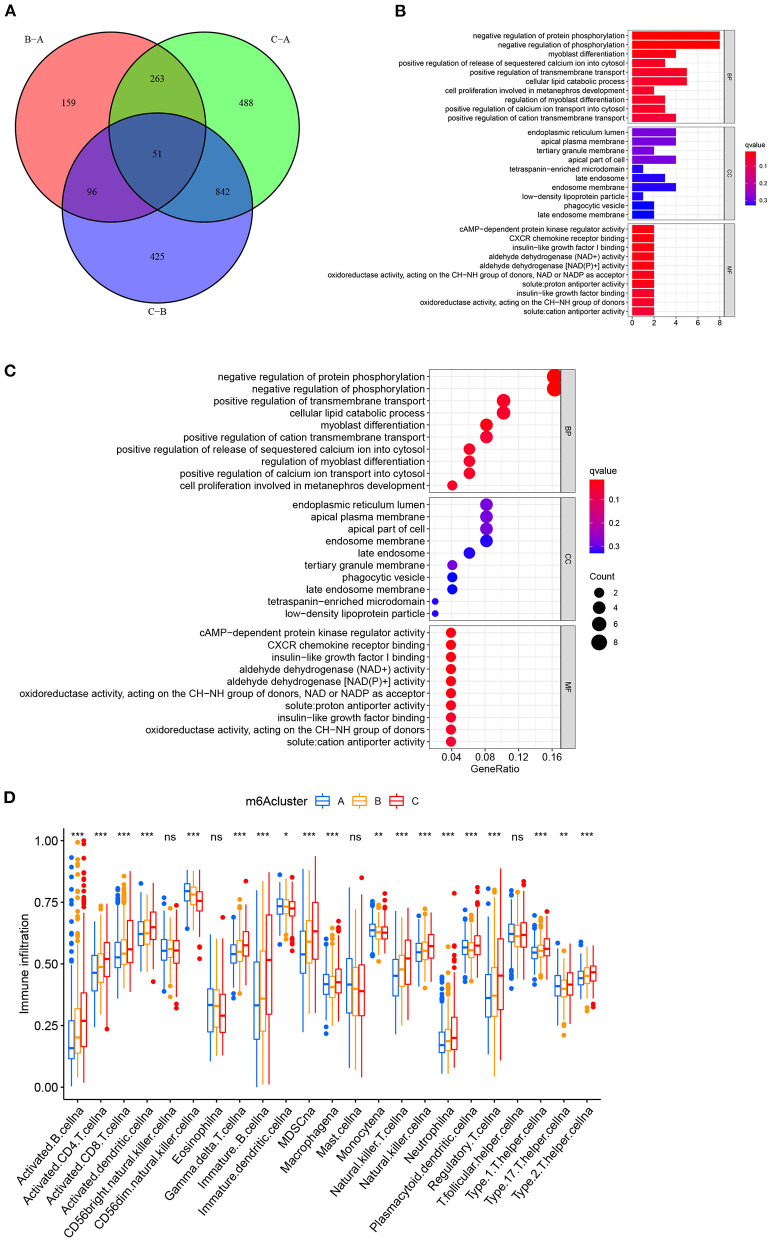
Functional enrichment analysis between three distinct m6A clusters in the merged cohort. **(A)** Venn diagram showing intersecting DEGs among three m6A clusters. (|logFC| > 1 and adjusted *p* < 0.01) **(B)** Gene Ontology enrichment analysis by a bar plot. **(C)** Gene Ontology enrichment analysis by a bubble chart. **(D)** ssGSEA analysis between three distinct m6A clusters based on immune-related gene expression. The asterisk symbol indicates the statistical *p*-value *p < 0.05; **p < 0.01; ***p < 0.001).

We first performed GSVA enrichment analysis to determine the different biological processes associated with the three distinct m6A clusters in bladder tumors. The supporting document used in GSVA analysis was shown in Supplementary Table 8. Figure 4A illustrated that m6A cluster A was significantly enriched in various metabolic biological processes, such as glycerophospholipid metabolism, arachidonic acid metabolism, endocytosis, and aldosterone-regulated sodium reabsorption. Compared to m6A cluster A, m6A cluster C was markedly enriched in various immune activation pathways, such as the NOD-like receptor signaling pathway, RIG-I-like receptor signaling pathway, antigen processing, and presentation pathway and intestinal immune network for IGA production. Compared to m6A cluster C, Figure 4B indicated that m6A cluster B was also significantly enriched in various metabolic biological processes, including glycerophospholipid metabolism, arachidonic acid metabolism, steroid hormone biosynthesis, retinol metabolism, metabolism of xenobiotics by cytochrome p450, and aldosterone-regulated sodium reabsorption. Both m6A cluster A and m6A cluster B for bladder tumors presented high metabolic characteristics. However, compared to m6A cluster A, the m6A cluster B was markedly enriched in various abnormal immune system activities, including systemic lupus erythematosus, graft vs. host disease and allograft rejection process (Figure 4C). DEG analysis between three m6A clusters was also performed. In total, 569 DEGs were identified comparing m6A cluster A with m6A cluster B, 1644 DEGs were identified comparing m6A cluster A with cluster C and 1414 DEGs were identified comparing m6A cluster B with cluster C. (|logFC| > 1 and adjusted *p* < 0.01) The Venn diagram in Figure 5A identified 51 intersecting DEGs among three distinct m6A clusters in the study. The intersecting DEGs were further used to conduct GO enrichment analysis. The GO enrichment results in the study were illustrated using a bar plot and bubble chart in Figures 5B,C. Various molecular functions, including protein phosphorylation, myoblast differentiation and ion transmembrane transport, were markedly enriched in GO analysis based on intersecting DEGs. To further explore the relationship between immune cells and different m6A modification clusters, ssGSEA was performed based on the immune-related gene expression files in Supplementary Table 9. Figure 5D indicated significant differences in almost all immune cells among the three m6A clusters in the study, which was consistent with the GSVA results. The m6A cluster C tended to obtain the highest immune scores of activated CD8 T cells, activated CD4 T cells, activated B cells and various antigen presentation process components, including aDCs, regulatory T cells, and Th2 cells. The m6A cluster A tended to exhibit the lowest immune scores for the above immune cells. Considering the best survival outcomes shown in Figure 3B, the high level of immune activity in m6A cluster C was believed to contribute to the survival advantage in this study. The list of DEGs discussed in this section was also supplied in Supplementary Table 10.

### Identification of Prognostic DEGs and the Development of m6A Gene Clusters

**Figure 6:**
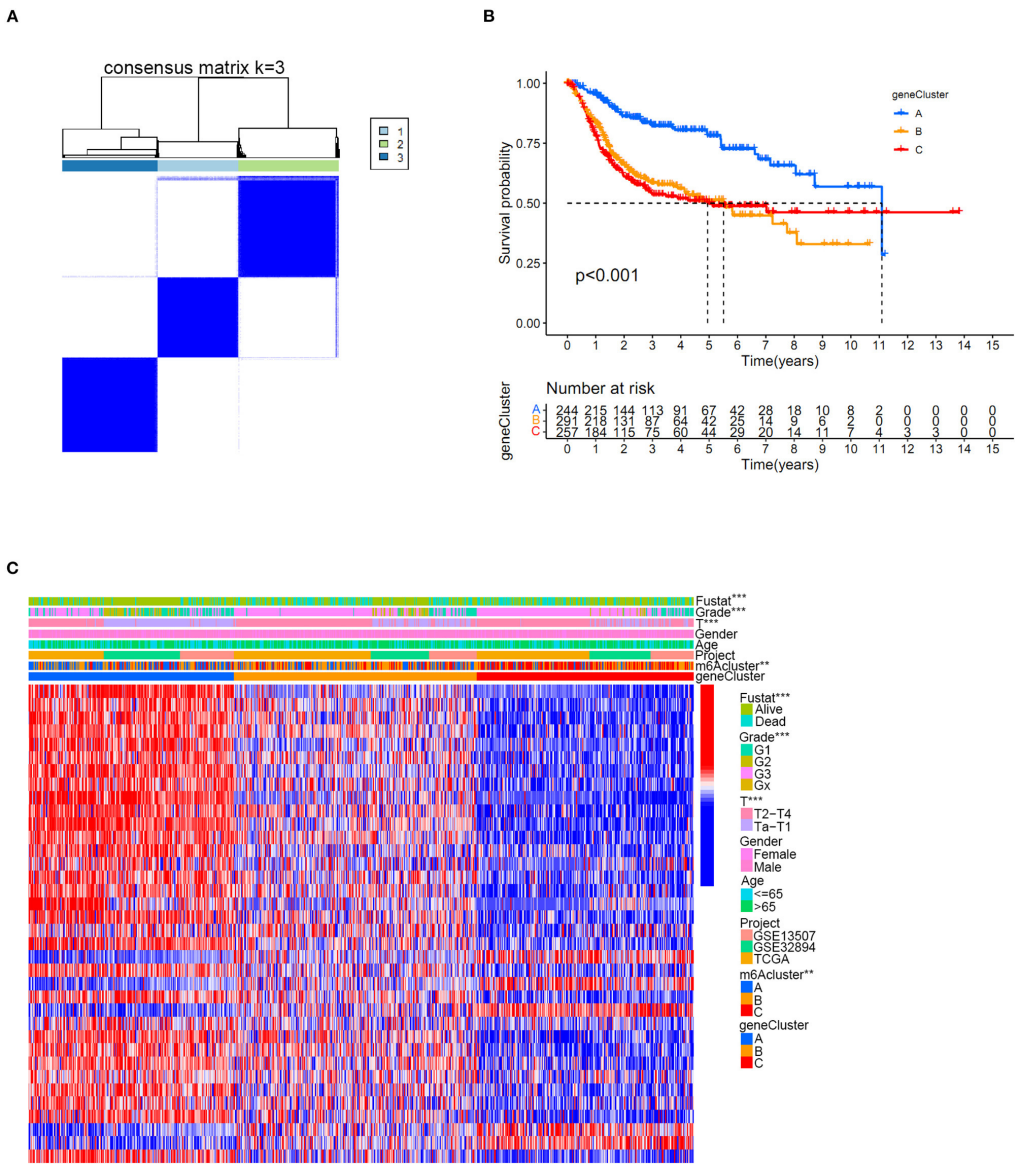
The m6A gene cluster results based on prognostic intersected DEGs in the merged cohort. **(A)** Consensus clustering matrix for *k* = 3. **(B)** Kaplan-Meier curve showing the survival prognostic value of three distinct m6A gene clusters. **(C)** Heatmap illustrating various clinicopathological features of three distinct m6A gene clusters (**p < 0.01; ***p < 0.001).

The univariate Cox regression method identified 36 prognostic genes from intersecting DEGs among three distinct m6A clusters. The detailed prognostic DEGs were listed in Supplementary Table 11, and their corresponding expression matrix in the merged cohort was illustrated in Supplementary Table 12. To further detail the m6A modification patterns in the merged cohort, an unsupervised clustering method depending on the expression of prognostic intersecting DEGs was then performed to explore more accurate m6A gene clusters. Then, k = 3 was finally selected as the optimal choice, and three clusters were finally determined, which we further named m6A gene clusters to separate them from our previous m6A clusters (Figure 6A). The m6A gene clusters were derived from the basis of m6A clusters in the study, so its value was of great importance to explore the possible m6A modification patterns in the study. Detailed cluster results of all merged samples were shown in Supplementary Table 13. A total of 262 bladder tumor samples were grouped for cluster A, 329 samples were grouped for cluster B, and 310 samples were grouped for cluster C. Depending on m6A gene clusters and clinical outcomes of the merged cohort in the study, Kaplan-Meier analysis was further used to explore survival differences between m6A gene clusters. Figure 6B showed that there was a significant difference in overall survival outcomes among m6A gene clusters (*p* < 0.001). The m6A gene cluster A had a good prognosis compared to m6A gene clusters B and C. To completely demonstrate the distributions of m6A gene cluster results, a heatmap combining all relative clinical characteristics was also developed, as shown in Figure 6C. The m6A gene clusters posed significant relationships with tumor grade, “T” stages and patients' survival status in this study. Considering that the m6A gene cluster was developed indirectly from the 10 m6A modification regulators, the expression differences of m6A regulators among three m6A gene clusters were also explored in this study. Supplementary Figure 1 showed that 80% (8/10) of m6A regulators, including RBM15, YTHDC1, YTHDC2, YTHDF1, IGFBP2, IGFBP3, RBMX, and ALKBH5, significantly differed among distinct m6A gene clusters. These findings indicated promising research prospects for m6A modification in the merged bladder tumor cohort.

### Quantification of the m6Ascore and Its Relationship With Survival Status, m6A Clusters, m6A Gene Clusters and Immune Cell Infiltration in the Merged Cohort

**Figure 7:**
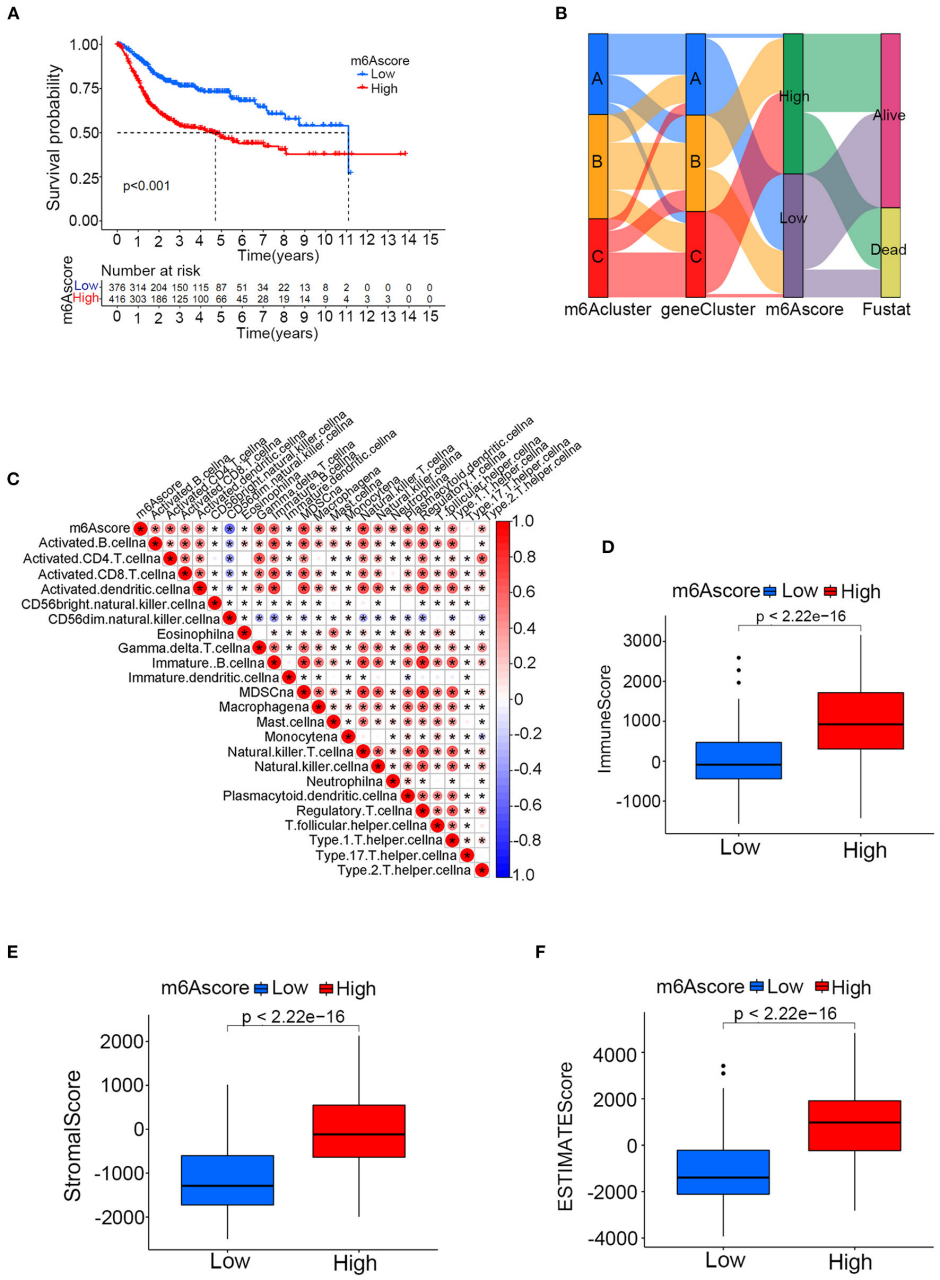
The relationships between the m6Ascore and m6A clusters, m6A gene clusters and various immune cell infiltration levels in the merged cohort. **(A)** Kaplan-Meier curve showing the survival prognostic value of the high/low m6Ascore groups. **(B)** Sankey diagram illustrating relationships among m6A clusters, m6A gene clusters and the m6Ascore in the merged cohort. **(C)** Correlation analysis between the m6Ascore and immune cell infiltrations. A negative correlation is indicated in blue, and a positive correlation is indicated red. The asterisk symbol indicates the statistical p-value. **(D–F)** Differences of immune score, stromal score and ESTIMATE score between high and low m6Ascore groups (*p < 0.05).

**Figure 8:**
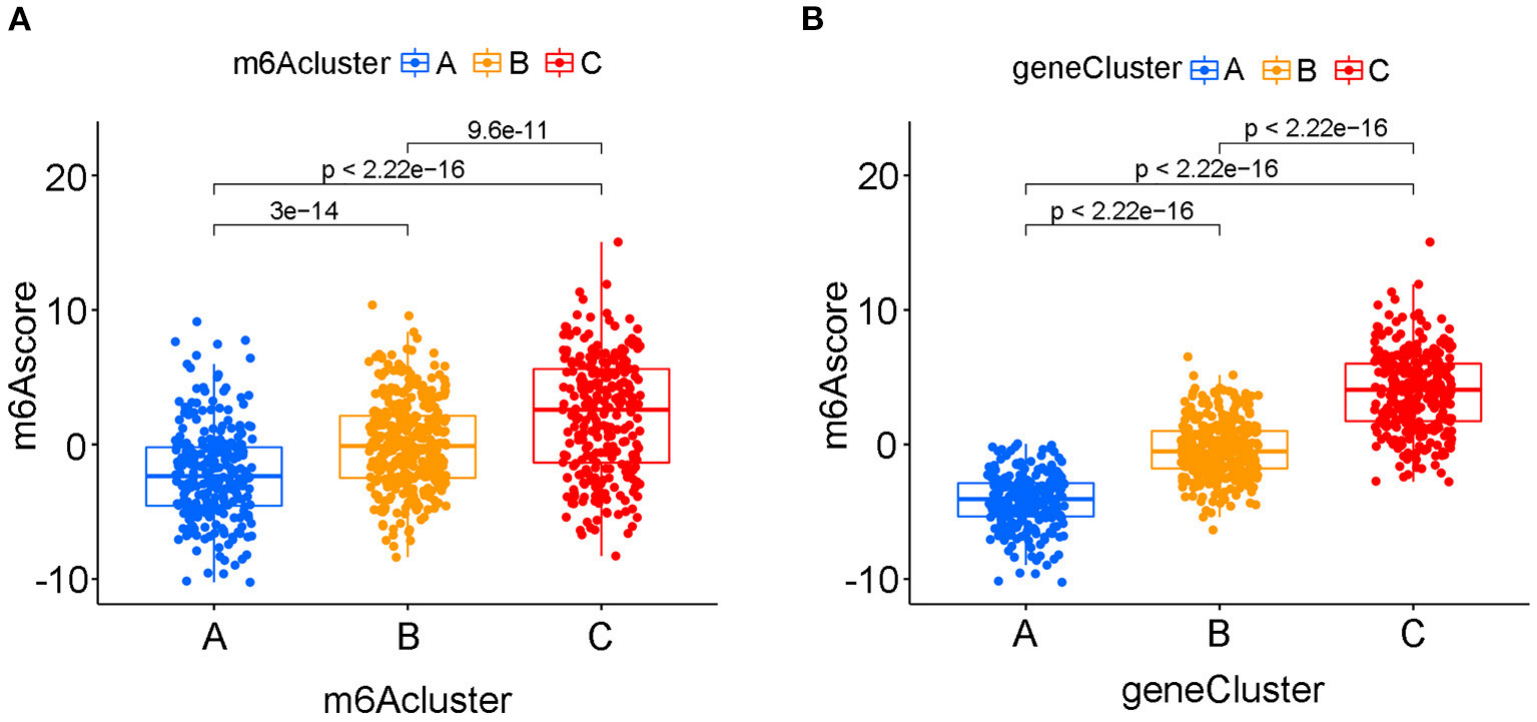
The quantifying difference of m6Ascore among different m6A clusters and different m6A gene clusters in merged cohort. **(A)** The quantifying difference of m6Ascore between three distinct m6A clusters. **(B)** The quantifying difference of m6Ascore between three distinct m6A gene clusters. The interquartile range of values was reprented by the lower and upper ends of boxes. Median value was represented by the lines during the boxes. Statistical difference was compared by Kruskal-Wallis test (*p* < 0.0001).

The above results confirmed that m6A modification played a vital role in grouping different clinical characteristics. However, these clusters, including both m6A clusters and m6A gene clusters, served as rough model exploration approaches in a large patient group. Quantified detailed m6A methylation modification levels were not identified for individual samples of bladder tumors. Then, the quantifying tool named the m6Ascore was developed using PCA based on the expression levels of prognostic intersected DEGs in the study. Supplementary Table 14 listed the detailed m6Ascore values for individual samples. The median m6Ascore in the merged study was utilized to classify the merged cohort into high or low m6Ascore groups (Supplementary Table 15). Figure 7A illustrated that the high m6Ascore group tended to exhibit a significantly worse survival prognosis than the low m6Ascore group. The Sankey diagram in Figure 7B showed interactive relationships between m6Ascore, m6A clusters, m6A gene clusters, and survival/death status. The various immune cell scores were supplied in Supplementary Table 16, and correlation analysis between the m6Ascore and immune cell scores was further conducted. Figure 7C showed that m6Ascore significantly correlated with almost all immune cells except CD56 natural killer cells and type 17 T helper cells. Figures 7D,E further demonstrated that high m6Ascore group tended to obtain significantly higher immune score and stromal score when compared with low m6Ascore patients. These significant correlations indicated that the m6A methylation modification patterns may interact closely with immune activities in organisms to contribute together to tumor occurrence. This study further explored m6Ascore differences between m6A clusters and m6A gene clusters. Figure 8A showed that m6A cluster C had the highest m6Ascore values, and m6A cluster A had the lowest m6Ascore values. Figure 8B illustrated that m6A gene cluster C had the highest m6Ascore values, and m6A gene cluster A had the lowest m6Ascore values. These results indicated that m6Ascore can be utilized to effectively distinguish different clusters in the study.

### The Correlation Between the m6Ascore and Gene Mutation Status

**Figure 9:**
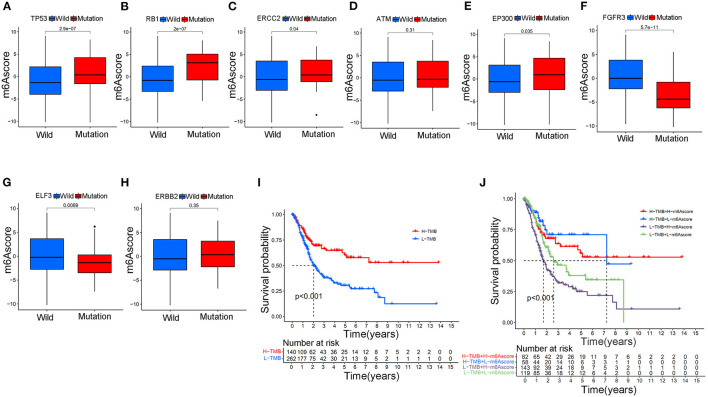
The relationship between the m6Ascore and gene mutation status in bladder tumors in the merged cohort. **(A)** The difference in the m6Ascore value between wild-type and mutant TP53 samples. **(B)** The difference in the m6Ascore value between wild-type and mutant RB1 samples. **(C)** The difference in the m6Ascore value between wild-type and mutant ERCC2 samples. **(D)** The difference in the m6Ascore value between wild-type and mutated ATM samples. **(E)** The difference in the m6Ascore value between wild-type and mutant EP300 samples. **(F)** The difference in the m6Ascore value between wild-type and mutant FGFR3 samples. **(G)** The difference in the m6Ascore value between wild-type and mutant samples of ELF3. **(H)** The difference in the m6Ascore value between wild-type and mutant ERBB2 samples. **(I)** Kaplan-Meier curve showing the prognostic value of survival in the high/low TMB groups in the merged cohort. **(J)** Kaplan-Meier curve showing the survival prognostic value of high/low TMB groups stratified by high/low m6Ascore in the merged cohort. The interquartile range of values was represented by the lower and upper ends of boxes. Median values are represented by the lines in the boxes.

The m6Ascore variations between different gene wild/mutation status of several classic responsible oncogenes (TP53, RB1, ERCC2, ATM, EP300, FGFR3, ELF3, ERBB2) were also explored in the study. Supplementary Table 17 summarized the mutation data of these oncogenes. Figure 9A illustrated that mutation of TP53 genes could significantly increase the m6Ascore values in the study (*p* < 0.001). Increasing m6Ascore was also identified among the RB1 mutation group (Figure 9B) (*p* < 0.001). The mutation of ERCC2 (*p* < 0.05) and EP300 (*p* < 0.05) also significantly increased the m6Ascore in the study. In contrast, mutation of FGFR3 (*p* < 0.001) and ELF3 (*p* < 0.01) significantly decreased the m6Ascore in this study. There was no difference identified for m6Ascore between the wild-type and mutant-type ATM and ERBB2 groups. These results indicated that the m6A methylation modification patterns were closely related to the activity of reported oncogenes for bladder tumors, such as TP53, RB1, ERCC2, EP300, FGFR3, and ELF3. To further explore the survival prediction significance of different mutation status and m6A high/low levels, we conducted survival analysis combining both TMB and m6A high/low levels in this study. The TMB information was uploaded in Supplementary Table 4. Figure 9I showed a good survival prognosis for patients in the high TMB group compared to those in the low TMB group. Figure 9J showed the survival prognosis analysis of TMB stratified by high/low m6Ascore levels. The groups with high TMB and low m6Ascore obtained the best survival prognosis in this study, followed by groups with high TMB and high m6Ascore and groups with low TMB and low m6Ascore. We surprisingly found that the groups with low TMB and high m6Ascore tended to obtain the poorest survival outcomes in the study (*p* < 0.001). Interestingly, no significant relationships between TMB and m6Ascore were identified in our Supplementary Figure 2.

### Relationships Between m6Ascore and Various Clinical Characteristics

**Figure 10:**
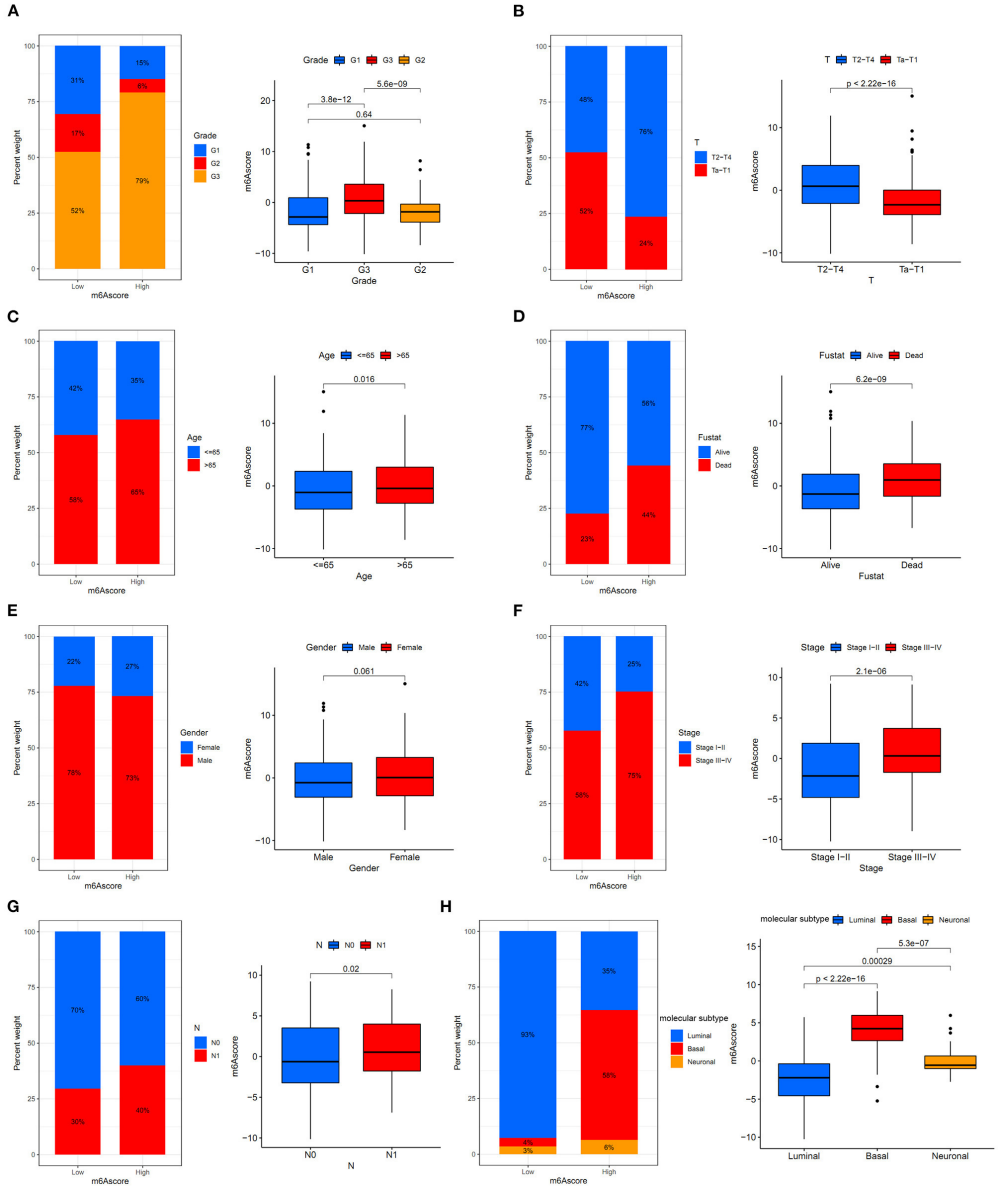
The relationships between the m6Ascore and various clinical characteristics. **(A)** Differential analysis of the m6Ascore among different tumor grades. **(B)** Differential analysis of the m6Ascore among different clinical stages. **(C)** Differential analysis of the m6Ascore among different aging patients. **(D)** Differential analysis of the m6Ascore among dead/alive groups. **(E)** Differential analysis of the m6Ascore among female/male groups. **(F)** Differential analysis of the m6Ascore among Stage III–IV and Stage I–II groups. **(G)** Differential analysis of the m6Ascore among N0/N1 groups. **(H)** Differential analysis of the m6Ascore among distinct molecular subtypes. Statistical differences were compared using the Kruskal-Wallis test (*p* < 0.0001). The interquartile range of values was represented by the lower and upper ends of boxes. Median values are represented by the lines in the boxes.

Figure 10A showed that the high m6Ascore group had a higher percent weight of G3 and lower percent weight of G1 and G2 than the low m6Ascore group. The bladder tumor samples with G3 also tended to obtain the highest m6Ascore quantification, and samples with G1 tended to obtain the lowest m6Ascore in the study (*p* < 0.001).

**Figure 11:**
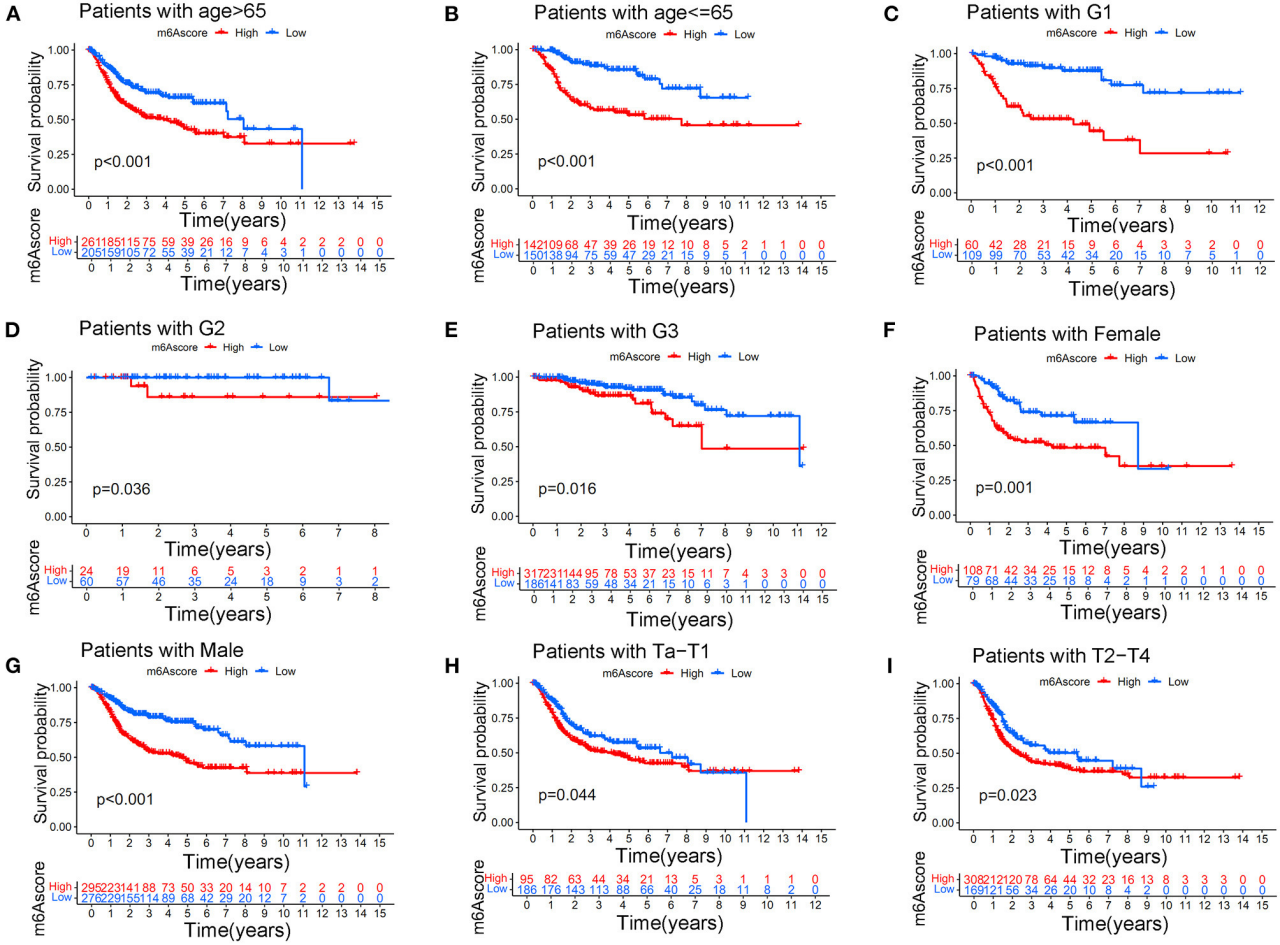
Kaplan-Meier curve showing the survival prognostic value of high/low m6Ascores in different stratified clinical groups. **(A)** Kaplan-Meier curve showing the prognostic value of high/low m6Ascores in patients aged > 65 years. **(B)** Kaplan-Meier curve showing the prognostic value of high/low m6Ascores in patients aged ≤ 65 years. **(C)** Kaplan-Meier curve showing the prognostic value of high/low m6Ascores in patients with G1 disease. **(D)** Kaplan-Meier curve showing the prognostic value of high/low m6Ascores in patients with G2 disease. **(E)** Kaplan-Meier curve showing the prognostic value of high/low m6Ascores in patients with G3 disease. **(F)** Kaplan-Meier curve showing the prognostic value of high/low m6Ascores in females. **(G)** Kaplan-Meier curve showing the prognostic value of high/low m6Ascores in males. **(H)** Kaplan-Meier curve showing the prognostic value of high/low m6Ascores on survival in patients with Ta-T1 stages. **(I)** Kaplan-Meier curve showing the prognostic value of high/low m6Ascores on survival in patients with T2-T4 stages.

This indicated that the m6Ascore in this study could be used to predict bladder tumor grade. Figure 10B also showed that the high m6Ascore group had a higher percent weight of T2-T4 and a lower percent weight of Ta-T1. Bladder tumor samples with T2-T4 stages also tended to have higher m6Ascore than those with Ta-T1 stages (*p* < 0.001). Figure 10C also indicated a higher percent weight of elderly samples (> 65 years old) in the high m6Ascore group. The m6Ascore levels were also significantly higher in the elderly population (*p* = 0.016). Figure 10D showed that the high m6Ascore group tended to have a higher percent weight of dead status, and a higher m6Ascore was positively correlated with worse prognosis for bladder cancer (*p* < 0.001), which was consistent with the prognosis analysis results shown in Figure 7A. Figure 10E indicated that there was no significant relationship identified between m6Ascore and sex in this study. Figure 10F demonstrated that tumors with Stage III–IV tended to obtain significantly higher m6Ascores when compared with Stage I–II. Patients with N1 status also indicated a higher m6Ascore when compared with N0 status (Figure 10G). An increasing number of researchers have suggested that bladder tumors should be classified into two or three molecular subtypes to improve the accurate prognosis in the clinic (22). We further explored potential relationships between m6Ascore and molecular subtypes, including the Luminal, Basal, and Neuronal subtypes in this study. The molecular subtypes for tumor samples in this study were available in Supplementary Table 18. Figure 10H showed that the high m6Ascore group had a significantly higher percent weight of the Basal subtypes. It also illustrated a significantly higher m6Ascore level during “Basal” subtypes compared to “Luminal” and “Neuronal” subtypes (*p* < 0.001). These results further confirmed the feasibility of the two-subtype classification method (“Luminal” and “Basal”). Furthermore, ROC analysis in Supplementary Figure 3 further demonstrated that m6Ascore posed the most excellent predictive role for survival prognosis when compared with clinical stage, tumor grade, and patients ages. A good quantification tool for bladder tumor patients should illustrate a consistent predictive role in different hierarchical classifications. Therefore, the prognostic value of the m6Ascore stratified by age, tumor grade, sex, and stage was also explored in the merged cohort. Figures 11A,B illustrated that the higher m6Ascore group had significantly poorer survival outcomes in both samples aged > 65 years old (*p* < 0.001) and samples aged ≤ 65 years old (*p* < 0.001). Figures 11C–E showed that a high m6Ascore still had prognostic value for survival outcomes in different groups stratified by G1 (*p* < 0.001), G2 (*p* < 0.05), and G3 (*p* < 0.05). The high m6Ascore also predicted poor survival prognosis in both female and male samples with *p* = 0.001 and *p* < 0.001 respectively (Figures 11F,G). The similar predictive roles of m6Ascore were also confirmed in both samples with Ta-T1 (*p* = 0.016) and samples with T2-T4 (*p* = 0.023) (Figures 11H,I).

### Potential Application of the m6Ascore for Guiding Immunotherapy Strategy Choices

**Figure 12:**
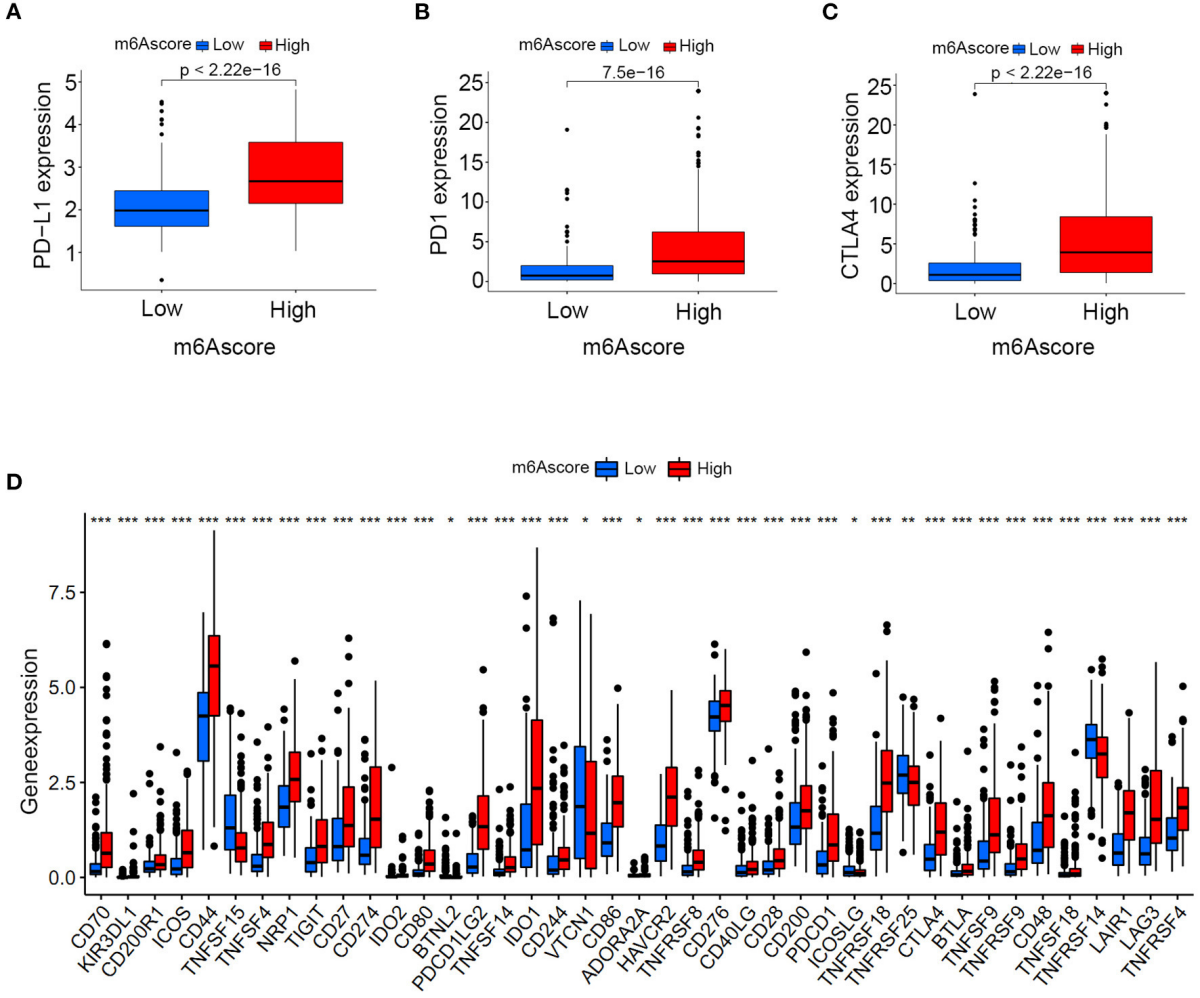
Expression analysis of immune checkpoints between the high and low m6Ascore groups. **(A)** PD-L1 expression differences between the high and low m6Ascore groups. **(B)** PD-1 expression differences between high and low m6Ascore groups. **(C)** CTLA-4 expression differences between high and low m6Ascore groups. **(D)** Expression differences of various immune checkpoints between high and low m6Ascore groups. The interquartile range of values was represented by the lower and upper ends of boxes. Median values are represented by the lines in the boxes.

**Figure 13:**
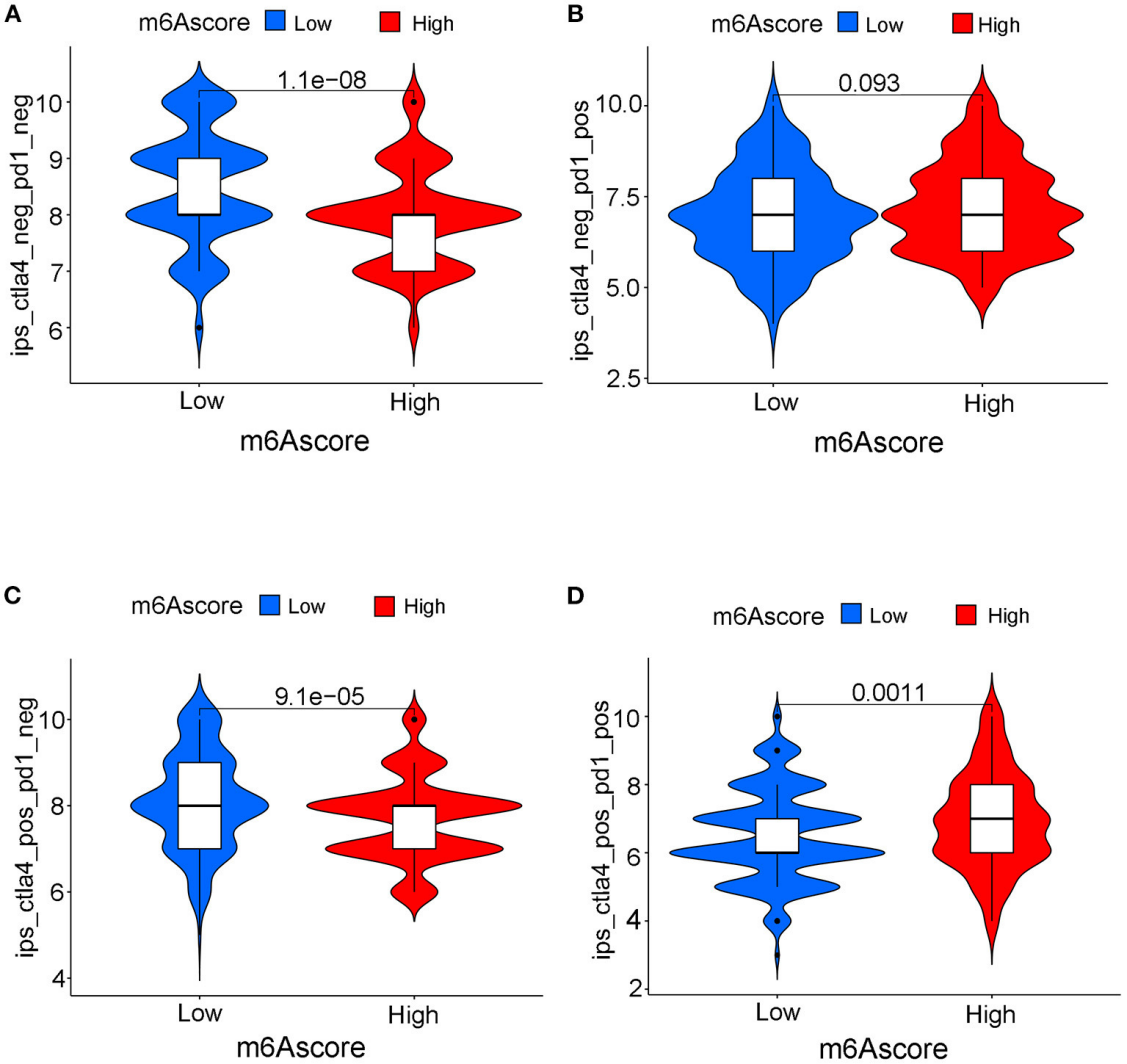
The predictive role of m6Ascore for different responses to immunotherapy strategies during immunotherapy cohort. **(A)** If none immunotherapy was conducted, the high m6Ascore resulted in a poor prognosis compared to low m6Ascore (*p* < 0.001). **(B)** If only anti-PD1 immunotherapy was used, there is no difference for therapeutic effects between high and low m6Ascore groups. **(C)** If only anti-CTLA4 immunotherapy was used, the higher m6Ascore group tended to get a poorer therapeutic effects compared to low m6Ascore group (*p* < 0.001) **(D)**. When anti-PD1 and anti-CTLA4 immunotherapy methods were simultaneously adopted, the high m6Ascore group might get significantly better prognosis compared to low m6Ascore group (*p* = 0.001).

Differences in expressions of various immune checkpoints were explored between the high/low m6Ascore groups in this study. Figure 12A illustrated that PD-L1 was significantly overexpressed in the high m6Ascore group (*p* < 0.001). Figure 12B indicated significantly higher PD-1 expression in the high m6Ascore group (*p* < 0.001). CTLA-4 was also significantly overexpressed in the high m6Ascore group, as shown in Figure 12C (*p* < 0.001). Figure 12D further confirmed that various immune checkpoints were differentially expressed between high and low m6Ascore groups. These results indicated a close relationship between m6Ascore and immune checkpoints. Furthermore, the immunotherapeutic cohort from http://tcia.at/ was further used to validate the relationship between the m6Ascore and immunotherapeutic response (Supplementary Table 19). If no immunotherapy was administered, the high m6Ascore resulted in a poor prognosis compared to the low m6Ascore. This result was consistent with previous survival analysis, as shown in Figures 7, 9. If only anti-PD1 immunotherapy was used, no difference in therapeutic effects was noted between the high and low m6Ascore groups. If only anti-CTLA4 immunotherapy was used, the higher m6Ascore group tended to obtain poorer therapeutic effects than the low m6Ascore group. When anti-PD1 and anti-CTLA4 immunotherapy methods were simultaneously administered, the high m6Ascore group exhibited a significantly better prognosis than the low m6Ascore group (*p* = 0.001) (Figure 13). Furthermore, drug sensitivity analysis using IC50 indicated that high m6A groups were potentially sensitive to various medical treatments including Bleomycin, Bortezomib, Cisplatin, Cyclopamine, Dasatinib, Docetaxe, Rapamycin, and Vinblastine (Supplementary Figure 4).

## Discussion

A total of 90–95% of urothelial carcinomas were pathologically confirmed as bladder tumors. Seventy-five percent of bladder tumors were transitional cell carcinomas, and 25% were other histology variants that posed great challenges to the current management of bladder tumors (25). Seventy percent of bladder tumors were classified as non-muscle-invasive tumors (NMIBCs), and 30% were classified as muscle-invasive tumors (MIBCs). Transurethral resection was currently suggested for NMIBC, and radical cystectomy was suggested for MIBC depending on AUA or EAU guidelines (26, 27). For advanced or metastatic bladder tumors, an increasing number of studies have suggested cisplatin-based cytotoxic chemotherapy to improve patient prognosis in the long term (28). In recent years, various advanced treatments, including various immunotherapy strategies, have also been suggested as second-line or first-line choices for patients who are ineligible to receive cisplatin-based chemotherapy (29). Various classification methods have previously been utilized to group bladder tumors into different stages or therapy responses depending on different clinical aspects (30). The most common term was the high or low grade depending on the classic pathological characteristic suggested by the WHO. Clinical stage Ta or T1 is referred to as NMIBC, and T2-T4 stages are defined as MIBC. Different treatment strategies for bladder tumors, including surgical interventions and novel adjuvant therapies, have been adopted according to different grades or stages; however, current therapies for bladder tumors are not able to prevent further tumor recurrence and metastasis. Approximately 21% of high-risk NMIBC progresses to MIBC according to recent studies (26). To further explore the molecular mechanism of the above classifications and to further improve the survival prognosis of bladder tumors, many mechanistic studies, including assessments of genetic alterations and molecular subtypes, have been conducted in recent years. The most reported oncogenes responsible for bladder tumors included P53, RB1, ERBB2, and FGFR3. Signature of molecular subtypes, including Basal and Luminal, were also developed from the mRNA expression analysis to improve the predictive and therapeutic implications (31). Both genetic alterations and molecular subtypes could play an important role in the therapeutic implications of bladder cancer. However, responses to current immunotherapy or chemotherapy were different, and these responses could not be effectively predicted by current classifications for bladder tumor. More detailed mechanistic studies including various posttranscriptional modifications are ongoing.

The m6A methylation modification was recently reported as an important posttranscriptional modification in organisms (32). An increasing number of studies have reported that the abnormal activities of m6A methylation could directly lead to the metabolism of downstream RNAs and proteins. Various types of malignant tumors have been reported to exhibit a close relationship with m6A methylation activities (33–35). However, whether m6A methylation modification played an inhibiting or promoting role in malignant tumors remained unknown. Some studies illustrated that the overexpression of m6A regulators, such as ALKBH5, could lead to a significant survival improvements (36). In addition, some studies also indicated that ALKBH5 overexpression triggered the development and invasion of malignant glioma by increasing FOXM1 expression products (34). METTL3 was also reported to play different inhibiting or promoting roles in different tumors. Wang et al. reported an oncogenic role of METTL3 in acute leukemia and liver tumors (37), and other studies also reported a tumor suppressor role of METTL3 in cervical cancer (38). Recently, studies also reported that ALKBH5 suppressed bladder cancer cell proliferation and induced sensitivity to cisplatin-based chemotherapy through m6A-CK2a-mediated glycolysis (39). These results have confirmed that the dysregulation of m6A methylation played a vital role in tumor development. However, the different roles of m6A regulators indicated a complex mechanism of methylation in organisms. Further exploration of the roles of m6A methylation modification in malignant tumors seemed to provide valuable information for future research on bladder cancer tumors. However, almost all previous studies concerning m6A methylation regulation have focused exclusively on one m6A regulator or one intrinsic carcinogenic pathway in recent years. The detailed procedure of m6A methylation was likely promoted by the interaction of various m6A regulators in organisms. A systemic study based on large cohorts was urgently needed to identify the detailed regulatory mechanism of m6A methylation patterns. In this study, we systematically explored the potential roles of m6A methylation in bladder tumors. We surprisingly identified that m6A methylation modification played a vital role in various characteristics of bladder tumors and could be utilized to predict the grade, stage, molecular subtypes and survival status from the perspective of posttranscriptional modification patterns. We quantified m6A modifications to directly evaluate the methylation level in each individual in our study. Further analysis in our study illustrated the predictive and promising guiding significance of these quantified m6A methylation levels in the study. The quantified m6A levels were referred to as the m6Ascore in the study and we identified that m6Ascore could be used to evaluate tumor prognosis, clinical stage, immune cell infiltration status, classic oncogene mutations, and molecular subtypes. The ROC analysis also demonstrated that m6Ascores posed the most excellent predictive roles for patients' survival outcomes when compared various clinical characteristics. More importantly, the m6Ascore could provide valuable guidance for different immunotherapy strategies in the merged cohorts.

Most readers, including YTHDC1, YTHDF3, HNRNPC, FMR1, LRPPRC, HNRNPA2B1, IGFBP3, and IGFBP1, tended to obtain an amplification of CNV, whereas all two erasers (FTO and ALKBH5) tended to obtain a depletion of CNV in bladder tumors. These results directly indicated an enhanced activity of the m6A methylation procedure in bladder tumors. Differential expression analysis of m6A methylation regulators identified significantly different expression levels between tumor and non-tumor samples. However, some m6A regulators, including METTL3, RBM15B, YTHDF1, YTHDF2, and IGFBP3, were significantly overexpressed in bladder tumors, and some m6A regulators, including METTL14, METTL16, ZC3H13, and YTHDF3, were markedly downregulated in bladder tumors. This result further showed that the occurrence of bladder tumors was not caused by a unified increase in m6A regulators but by the interaction cooperation between various regulators. In this study, 24.51% of the samples exhibited genetic alterations in m6A methylation regulators, and this percentage also indicated an important role of m6A methylation activities in bladder tumors. Furthermore, the most responsible genetic alterations in this study included METTL3, RBM15, YTHDC2, LRPPRC, and ZC3H13. More experimental verification depending on these regulators is needed in the future. Figure 2A illustrated the interaction network of different m6A regulators in the merged cohort. Every gene posed an interactive correlation with each other. ALKBH5 was identified as an important cross-linked gene in the network. Yang et al. reported that ALKBH5 overexpression improved patient prognosis, and Cheng et al. also reported an inhibitory role of ALKBH5 in bladder tumors (15, 16). However, this study indicated an oncogenic role of ALKBH5 in bladder tumors (*p* < 0.01), which was consistent with various studies that showed that ALKBH5 overexpression significantly induced malignant tumors (40, 41). The different roles of ALKBH5 noted in different studies were believed to be caused by the inclusion of different samples in the studies. Our study included 901 bladder tumors to comprehensively analyze the m6A modification patterns, and the risk role of ALKBH5 was considered valuable for future mechanistic studies. Seventy percent of m6A regulators in Figure 2 exhibit significant predictive roles for patient survival in the merged cohort, further indicating the important role of m6A methylation in bladder tumors.

We constructed two different cluster methods, including the m6A cluster and m6A gene cluster, using unsupervised clustering analysis. The transformation procedure from m6A clusters to m6A gene clusters was a necessary and essential procedure to improve the accurate prognostic ability of m6A methylation modification patterns. First, m6A clusters A and B were both significantly enriched in various metabolic biological processes, and m6A cluster C was significantly enriched in various immune activation pathways. Further ssGSEA analysis also confirmed that m6A cluster C tended to exhibit the highest immune scores of activated CD8 T cells, activated CD4 T cells, and activated B cells, and m6A cluster A tended to obtain the lowest immune scores for the above immune cells. Previous studies have confirmed that high immune cell infiltration was positively correlated with good prognosis for malignant tumors (42). Figure 3B illustrated a good prognosis for the high immune infiltration cluster-m6A cluster C, which was consistent with previous research results (42). However, the low immune infiltration cluster A exhibited better prognosis compared with m6A cluster B in this study. Further GSVA analysis revealed that m6A cluster B was markedly enriched in various abnormal immune system activities, including systemic lupus erythematosus, graft vs. host disease and allograft rejection processes. The enrichment of various abnormal immune activities was believed to be responsible for the poor prognosis noted in m6A cluster B. Notably, Figure 8A showed that m6Ascore of m6A cluster B was lower than that of cluster C, however, the m6A cluster B posed significant poorer prognosis than cluster C (Figure 3B). This result also indicated that m6A clusters posed inconsistent roles in predicting prognosis with m6Ascores. Therefore, the replacement of m6A clusters by m6A gene clusters was necessary to more accurately explore the role of m6A modification patterns in our study. Furthermore, the m6Ascore in this study was identified to increase progressively from m6A gene clusters A to C. The survival analysis results of both the m6Ascore (Figure 7A) and m6A gene clusters (Figure 6B) both indicated a risk- factor predictive role of the high m6Ascore (*p* < 0.01) or m6A gene cluster C (*p* < 0.01). The predictive role of m6A modification patterns was highly consistent in m6A and m6A gene cluster analysis.

Regarding relationships between the m6Ascore and various clinical characteristics, this study revealed that higher tumor grade (*p* < 0.01), more advanced clinical stages (*p* < 0.01), elder age (*p* = 0.016), Stage III–IV (*p* < 0.01), and N1 status (*p* = 0.02) tended to be quantified as higher m6Ascores. There was no difference for m6Ascore between different genders. These results directly indicated that bladder tumors with higher tumor grade, more advanced clinical stages and elder ages often experienced overactive m6A methylation modification in bladder tumors. The m6Ascore may be used as a valuable tool for tumor progression evaluation from a more mechanistic perspective. The survival analysis in Figure 11 further confirmed the excellent feasibility of m6Ascore to evaluate survival prognosis in different subgroups.

The TMB of bladder tumors ranked fourth according to previous publishes, following melanoma and lung cancers (43). High TMB contributed to an increased neoantigen burden for bladder tumors, which could greatly improve the therapy response to immunotherapy (44, 45). High TMB could significantly improve the survival prognosis of bladder tumors treated with immunotherapy. The relationship between the m6Ascore and gene mutation status was also explored in this study. TP53 (*p* < 0.001), RB1 (*p* < 0.001), ERCC2 (*p* < 0.05), and EP300 (*p* < 0.05) mutations significantly increased the m6Ascore evaluation, whereas FGFR3 (*p* < 0.001) and ELF3 (*p* < 0.01) mutations significantly downregulated m6Ascore evaluation in this study (Figure 9). These results indicated that the m6A methylation modification patterns were closely related to the activity of the above reported oncogenes in bladder tumors. The high TMB and low m6Ascore groups exhibited the best survival prognosis in this study followed by the high TMB and high m6Ascore groups and the low TMB and low m6Ascore groups. Groups with low TMB and high m6Ascores tended to exhibit the poorest survival outcomes in the study (*p* < 0.001). These results were consistent with previous studies that reported a good prognosis for high TMB status (44, 45). Interestingly, Supplementary Figure 2 demonstrated that there were no internal relationships between m6Ascore and TMB in bladder cancers. Strategies combining m6Ascore and TMB together could help to comprehensively evaluate prognosis outcomes for bladder cancers. With the development of mRNA sequencing technology in recent years, molecular subtypes of bladder tumors have also been reported in various studies (46, 47). Some studies defined two molecular subtypes (Luminal and Basal), whereas other studies defined three molecular subtypes (Luminal, Basal and Neuronal) for bladder tumors. This study identified a significant difference in m6Ascores between different reported molecular subtypes (Figure 10H). The Basal subtype tended to obtain the highest m6Ascore quantification, and the Luminal subtype tended to obtain the lowest m6Ascore quantification. Our results further confirmed the feasibility of the two molecular subtype classification methods in previous studies. Choi et al. reported that the Luminal subtype of bladder tumors was positively correlated with lower grade and better survival prognosis (46). The low m6A score for Luminal subtypes and high m6A score for Basal subtypes observed in our study also confirmed previous research results regarding m6A regulation.

An increasing number of studies have reported a close relationship between the expression of immune checkpoints and tumor gene mutation load in various tumor types, including bladder, melanoma, breast, and cervical cancer (35). PD-L1, PD-1, and CTLA4 played vital roles in immune-surveillance and were often up-regulated in malignant cells (48). PD-L1/PD-1 overexpression was usually associated with treatment failure and poor survival prognosis in bladder tumors. The evaluation of PD-L1/PD-1 expression is currently used to identify patients who are suitable for immunotherapy. The relationship between the m6Ascore and TMB was confirmed in the above analysis, and its relationship with immune checkpoints was also explored in this study. Figure 12 illustrated significantly up-regulated levels of PD-L1 (*p* < 0.001), PD-1 (*p* < 0.001), and CTLA-4 (*p* < 0.001) among the high m6Ascore groups. These results indicated that the m6Ascore in our study can also be used to evaluate the expression levels of immune checkpoints. PD-L1/PD1 up-regulation acts as a mechanistic target for immunotherapy and has been proven to be effective in preventing tumor progression (49). However, responses to immunotherapy greatly vary in different individuals due to the complex tumor environment. Various primary and secondary resistances lead to a poor response to immunotherapy. There were no robust biomarkers that could be utilized to predict clinical response or benefit for immunotherapy at this time. The different responses to immunotherapy are attributed to different factors, including both patient and tumor variables. Only usage of PD-L1/PD-1 expression is not able to accurately predict survival benefit according to previous studies (49). An increasing number of clinical trials involving various combinations of immunotherapies are currently being assessed for bladder tumor treatment to improve survival benefit (45, 50). The analysis performed in this study illustrated the good predictive role of the m6Ascore for survival benefit among different immunotherapy strategies. If only anti-PD1 immunotherapy was used, no difference in therapeutic effects was noted between the high and low m6Ascore groups. If only anti-CTLA4 immunotherapy was used, the higher m6Ascore group tended to exhibit poorer therapeutic effects than the low m6Ascore group. When anti-PD1 and anti-CTLA4 immunotherapy were simultaneously administered, the high m6Ascore group exhibited a significantly better prognosis than the low m6Ascore group. Our study further predicted potential medical treatments which high m6Ascore groups might be sensitive to. Given the various predictive roles for tumor grade, clinical stages, survival prognosis, molecular subtypes, gene mutation load, PD-L1/PD-1 expression levels, and treatment benefits for different immunotherapy strategies, the m6Ascore described in this study robustly confirmed an important role of m6A methylation modification patterns in bladder tumors, and this information could be utilized to guide immunotherapies in the future.

There were also several limitations in this study. Firstly, all analyzed samples were derived from public datasets and more genetic sequencing information from multi-centers were needed in further validation. Secondly, owing to different platforms of TCGA, GSE13507 and GSE32894, only information including age, tumor grade, T stage, survival outcomes, and gender were available in merged cohort. The clinical stages, lymph node metastasis, and molecular subtypes were analyzed only in TCGA cohort. More samples with available information were needed to explore relationships between m6A modification patterns and various characteristics. Furthermore, molecular mechanism underlining m6A modifications in bladder cancer development needed to be investigated by further experimental validations.

## Conclusions

This study systematically analyzed the important roles of m6A methylation modification patterns in bladder tumors. Different m6A modification patterns were closely related to tumor grade, clinical stage, survival prognosis, molecular subtype, gene mutation load, PD-L1/PD-1 expression levels and treatment benefits for different immunotherapy strategies. Detailed quantification of m6A modification patterns could improve our understanding of the bladder tumor microenvironment and could provide guidance for future immunotherapy strategies.

## Data Availability Statement

The datasets presented in this study can be found in online repositories. The names of the repository/repositories and accession number(s) can be found in the article/Supplementary Material.

## Author Contributions

JL conducted data collection and data analyzing procedure. JianW, WZ, LM, and JiawW conducted data collection. ZL, HX, and MW revised figures and documents. JW and YZ designed the study. All authors approved the final submitted version for this document.

## Conflict of Interest

The authors declare that the research was conducted in the absence of any commercial or financial relationships that could be construed as a potential conflict of interest.

## Publisher's Note

All claims expressed in this article are solely those of the authors and do not necessarily represent those of their affiliated organizations, or those of the publisher, the editors and the reviewers. Any product that may be evaluated in this article, or claim that may be made by its manufacturer, is not guaranteed or endorsed by the publisher.

## Supplementary Material

Supplementary Table 1 List of m6A methylation modification regulators.

Supplementary Table 2 Copy number variations in m6A regulators.

Supplementary Table 3 The gene mutation files of the TCGA cohort.

Supplementary Table 4 The calculated tumor mutation burden of TCGA cohort.

Supplementary Table 5 mRNA expression of m6A modification regulators in the merged cohort.

Supplementary Table 6 Detailed m6A cluster results of the 901 samples in the merged cohort.

Supplementary Table 7 Detailed clinical information for the merged cohort.

Supplementary Table 8 The referenced document used for GSVA analysis.

Supplementary Table 9 The referenced document used for ssGSEA.

Supplementary Table 10 The 51 intersecting DEGs among three distinct m6A clusters in the merged cohort.

Supplementary Table 11 The 36 prognostic intersecting DEGs identified from the univariate Cox analysis of factors associated with survival status in the merged cohort.

Supplementary Table 12 Detailed expression matrix of the 36 prognostic intersecting DEGs in the merged cohort.

Supplementary Table 13 Detailed m6A gene cluster results of the 901 samples from the merged cohort.

Supplementary Table 14 The m6A score quantification results of the 901 bladder tumors in the merged cohort.

Supplementary Table 15 High/low grouping results of the m6Ascore based on the median m6Ascore for the merged cohort.

Supplementary Table 16 Detailed infiltrating scores for immune cells in the merged cohort.

Supplementary Table 17 Detailed gene mutation status of classical oncogenes for bladder tumors.

Supplementary Table 18 Detailed molecular subtypes of individual bladder tumors from the study.

Supplementary Table 19 Detailed immunotherapy response scores for four different immunotherapy strategies.

Supplementary Figure 1 Differences in the expression of m6A modification regulators among three distinct m6A gene clusters. The asterisk symbol indicates the statistical *p*-value (^*^*p* < 0.05; ^***^*p* < 0.001).

Supplementary Figure 2 **(A)** There was no difference of TMB value between high and low m6Ascore groups. **(B)** Correlation analysis between TMB and m6Ascore in this study.

Supplementary Figure 3 **(A–E)** ROC analysis concerning predictive abilities of various characteristics for survival prognosis from 1 to 5 years.

Supplementary Figure 4 Drug sensitivity analysis utilizing half maximal inhibitory concentration (IC50) between high and low m6Ascore groups. High m6A groups were potentially sensitive to various medical treatments including **(A)** Bleomycin, **(B)** Bortezomib, **(C)** Cisplatin, **(D)** Cyclopamine, **(E)** Dasatinib, **(F)** Docetaxe, **(G)** Rapamycin, and **(H)** Vinblastine.

## Abbreviations

TCGA: The Cancer Genome Atlas
GEO: Gene Expression Omnibus
KEGG: Kyoto Encyclopedia of Genes and Genomes
m6A: N6-methyladenosine modification
GO: Gene Ontology
PD-1: Immune Checkpoint Programmed Cell Death 1
ssGSEA: Single-sample Gene Set Enrichment Analysis
PD-L1: Immune Checkpoint Programmed Cell Death 1 Ligand
DEGs: Differentially Expressed Genes
CTLA-4: Cytotoxic T Lymphocyte Antigen 4
RB1: retinoblastoma 1
TP53: Tumor Protein 53
ERBB2: erb-b2 receptor tyrosine kinase 2
ERCC2: ERCC excision repair
FGFR3: Fibroblast Growth Factor Receptor 3
GSVA: Gene Set Variation Analysis
TMB: Tumor Mutation Burden
CNV: Copy Number Variations
PCA: Principal Component Analysis.
