# Supplementary figures and images for: Comprehensive Analysis of N6-methyladenosine Modification Patterns Associated With Multiomic Characteristics of Bladder Cancer
Source: Front Med (Lausanne). 2021 Dec 23;8:757432. doi: 10.3389/fmed.2021.757432 (PMC8733309; doi:10.3389/fmed.2021.757432)

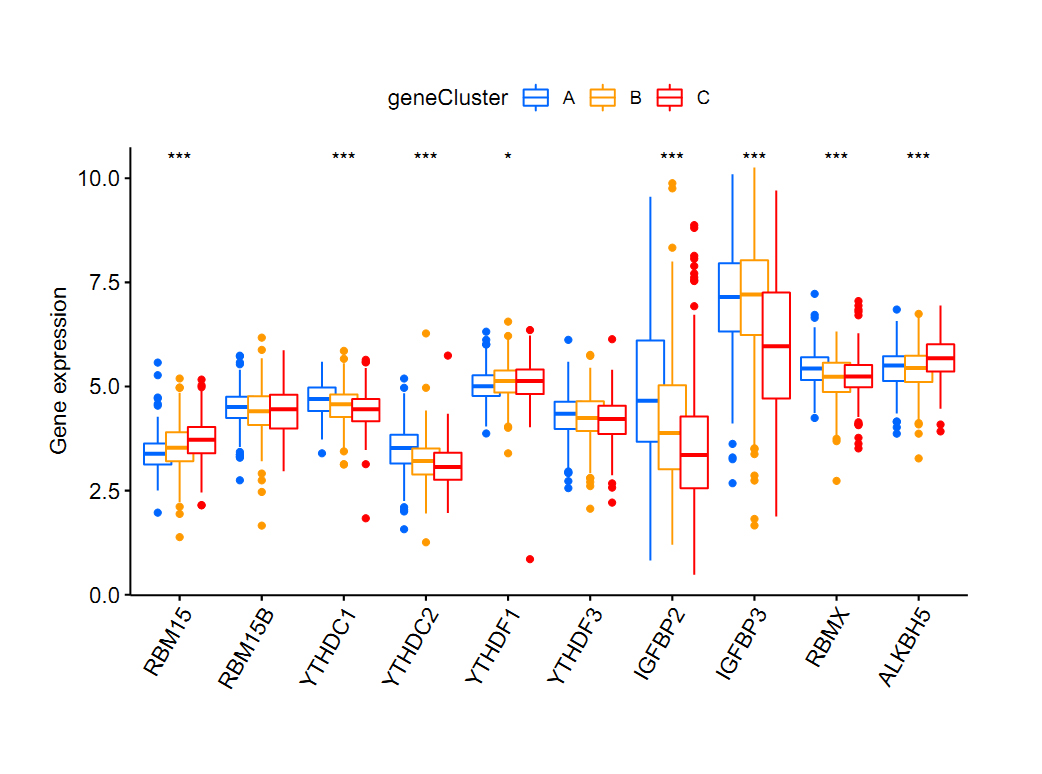

Supplement: Supplementary Figure 1 — Differences in the expression of m6A modification regulators among three distinct m6A gene clusters. The asterisk symbol indicates the statistical p-value (*p < 0.05; ***p < 0.001). [file Image_1.JPEG]

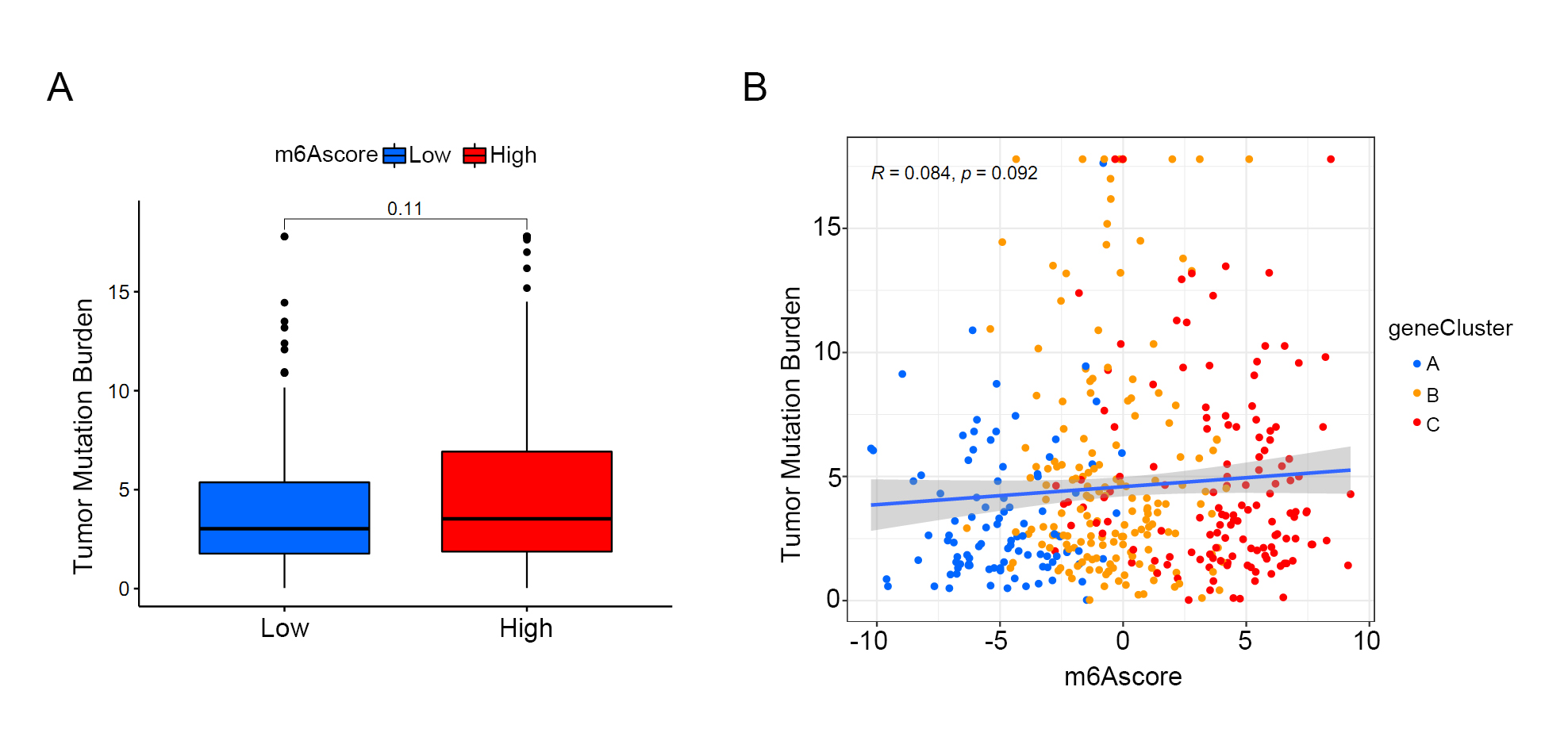

Supplement: Supplementary Figure 2 — (A) There was no difference of TMB value between high and low m6Ascore groups. (B) Correlation analysis between TMB and m6Ascore in this study. [file Image_2.JPEG]

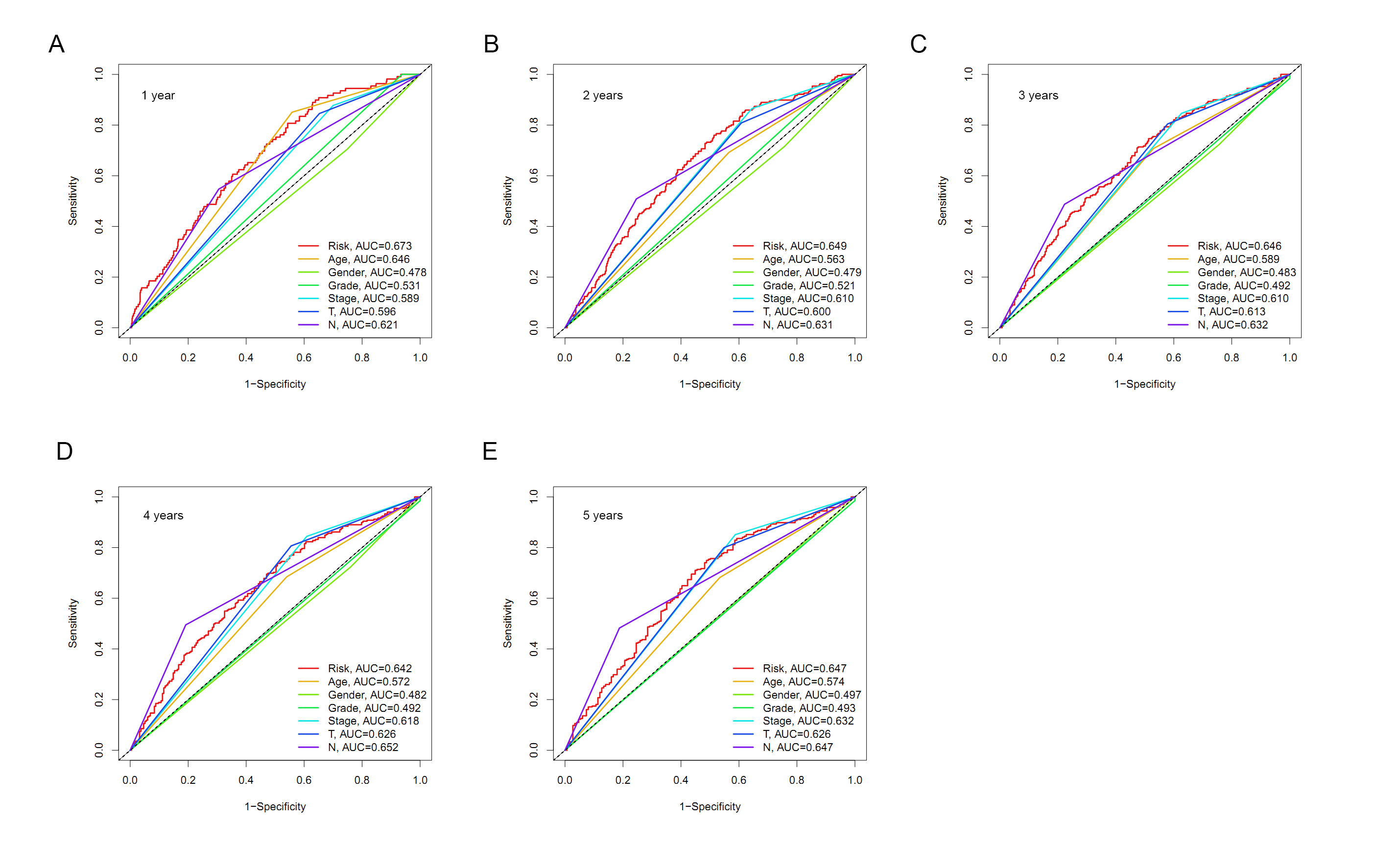

Supplement: Supplementary Figure 3 — (A–E) ROC analysis concerning predictive abilities of various characteristics for survival prognosis from 1 to 5 years. [file Image_3.JPEG]

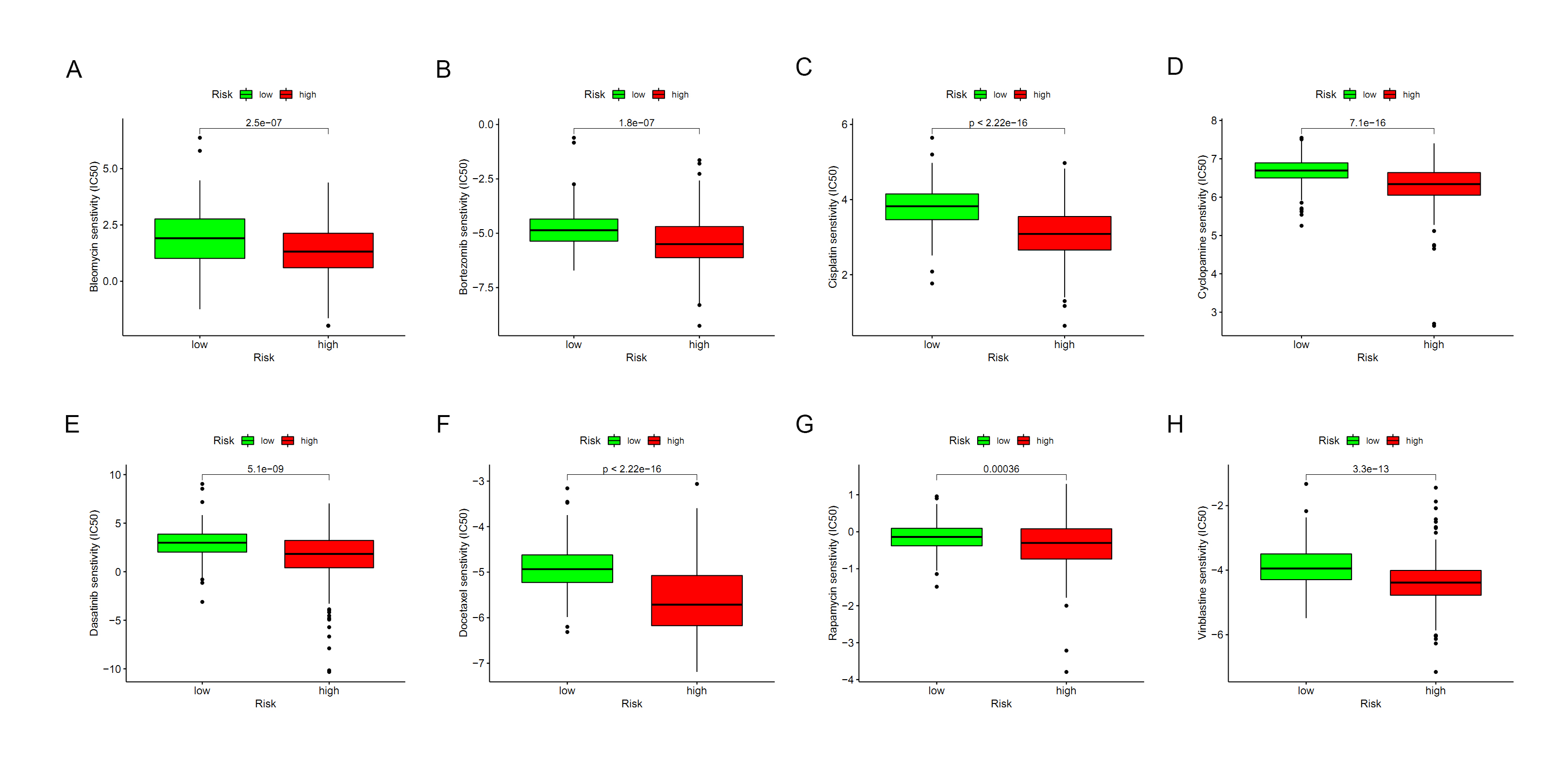

Supplement: Supplementary Figure 4 — Drug sensitivity analysis utilizing half maximal inhibitory concentration (IC50) between high and low m6Ascore groups. High m6A groups were potentially sensitive to various medical treatments including (A) Bleomycin, (B) Bortezomib, (C) Cisplatin, (D) Cyclopamine, (E) Dasatinib, (F) Docetaxe, (G) Rapamycin, and (H) Vinblastine. [file Image_4.JPEG]
